# Neutrophil extracellular trap components and myocardial recovery in post-ischemic acute heart failure

**DOI:** 10.1371/journal.pone.0241333

**Published:** 2020-10-29

**Authors:** Miriam Sjåstad Langseth, Geir Øystein Andersen, Trygve Husebye, Harald Arnesen, Manuela Zucknick, Svein Solheim, Jan Eritsland, Ingebjørg Seljeflot, Trine Baur Opstad, Ragnhild Helseth

**Affiliations:** 1 Center for Clinical Heart Research, Oslo University Hospital Ullevål, Oslo, Norway; 2 Faculty of Medicine, University of Oslo, Oslo, Norway; 3 Department of Internal Medicine, Drammen Hospital, Vestre Viken HF, Drammen, Norway; 4 Department of Cardiology, Oslo University Hospital Ullevål, Oslo, Norway; 5 Oslo Centre for Biostatistics and Epidemiology, Institute for Basic Medical Sciences, University of Oslo, Oslo, Norway; Kurume University School of Medicine, JAPAN

## Abstract

**Objective:**

The role of neutrophil extracellular traps (NETs) in acute heart failure is unknown. We recently showed that interleukin 8, a putative NETs stimulator, was associated with myocardial recovery in acute heart failure complicating ST-elevation myocardial infarction (STEMI). In this exploratory post-hoc study, we aimed to investigate the role of NETs components in relation to myocardial function and interleukin 8 in STEMI patients with symptomatic acute heart failure.

**Methods:**

In 61 STEMI patients developing acute heart failure within 48 hours of successful revascularization, wall motion score index (WMSI), global longitudinal strain (GLS) and left ventricular ejection fraction (LVEF) were assessed by echocardiography at baseline and on day 5. Blood drawn at baseline and days 1, 2 and 5 was used to quantify double-stranded DNA (dsDNA), myeloperoxidase-DNA complexes (MPO-DNA) and citrullinated histone 3 (CitH3). The area under the curve (AUC) of each NETs marker and interleukin 8 was approximated for the first 5 days.

**Results:**

dsDNA_AUC_ and MPO-DNA_AUC_ correlated significantly with change in WMSI from baseline to day 5 (r_s_ = 0.28 for both, p≤0.05), whereas NETs AUCs did not correlate with changes in GLS and LVEF. dsDNA_AUC_ was significantly correlated with interleukin 8_AUC_ (r = 0.40, p = 0.003). However, mixed model regression could not identify a significant effect of the NETs components on myocardial function parameters.

**Conclusions:**

In this cohort with acute heart failure complicating STEMI, NETs components were partly correlated with myocardial function and interleukin 8 levels, yet no causal relationship between NETs components and myocardial recovery could be established.

**Clinical trial registration:**

ClinicalTrials.gov, identifier: NCT00324766.

## Introduction

Although patient outcomes after ST-elevation myocardial infarction (STEMI) have improved considerably over the last decades, the incidence of heart failure during hospitalization for the index myocardial infarction (MI) is still estimated at nearly 30% [1], necessitating the ongoing search for biomarkers and potential therapeutic targets. In this pursuit, sustained inflammation has gained attention as a contributor to the development of ischemic heart failure [2–4].

Among the inflammatory mediators that are elevated post-MI, interleukin 8 (IL-8) attracts neutrophils that infiltrate the myocardium within hours of infarction and contribute to myocardial injury through recruitment of other inflammatory mediators, degranulation and production of reactive oxygen species [5]. We recently showed that increased levels of IL-8 were associated with poorer myocardial recovery in patients with acute heart failure (AHF) complicating STEMI treated by percutaneous coronary intervention (PCI) [6]. Aside from expulsion of reactive oxygen species and other proteins, neutrophils also release extracellular traps (NETs), appearing as extracellular clouds consisting of deoxyribonucleic acid (DNA), histones, and neutrophil granule proteins in response to stimuli such as activated platelets or IL-8 [7]. NETs have been shown to be cytotoxic, proinflammatory and prothrombotic, potentially augmenting myocardial injury [8,9], and have recently been suggested to affect both myocardial infarct size and post-MI left ventricular remodeling [10,11]. However, the contribution of NETs components with respect to AHF complicating MI has not previously been reported on.

We hypothesized that increased levels of circulating NETs components might reflect an unfavourable immune response as part of sterile inflammation post-MI, potentially affecting myocardial recovery after ischaemia and reperfusion (IR) injury. The present exploratory study therefore aimed to investigate whether NETs component levels were associated with echocardiographic indices of myocardial function in patients with AHF complicating STEMI. Secondarily, we explored whether there was any association between NETs components and circulating IL-8 levels as a putative stimulator of NETosis.

## Materials and methods

### Study design

Patients included in this post hoc observational sub-study (*n* = 61) were enrolled in the LEvosimendan in Acute heart Failure (LEAF) trial at the Oslo University Hospital Ullevål from 2006–2010. Results and details of the study design have previously been published [12]. In brief, patients with STEMI and clinical signs of heart failure within 48 hours of successful revascularization by PCI were randomized double blind to a 25-hour infusion of either levosimendan or placebo (S1 Fig). The primary endpoint was change in left ventricular wall motion score index (WMSI) from baseline to day 5. Secondary outcomes included changes in inflammatory markers and infarct size at six-week follow-up.

Clinical signs of heart failure were defined as shortness of breath at rest, and the presence of at least one of the following: pulmonary oedema, pulmonary congestion on chest x-ray, requiring mechanical ventilatory support or intravenous diuretics, or persistent oliguria (urine output <0.5 ml/kg/h) despite volume therapy. A subgroup of patients in cardiogenic shock were stratified by block randomization, and the inclusion criteria were systolic blood pressure <90 mmHg after one hour of adequate volume therapy, or systolic blood pressure 90–100 mmHg with ongoing inotropic treatment, in addition to clinical signs of hypoperfusion including oliguria, cold and clammy extremities, or reduced consciousness.

The study conforms with the principles outlined in the Declaration of Helsinki, and was approved by the South East Regional Ethics Committee, Norway. All participants gave written informed consent. The LEAF trial is registered with ClinicalTrials.gov, identifier: NCT00324766.

### Myocardial imaging

Echocardiography was performed by two experienced echocardiographers at baseline, on days 1 and 5, and at the 6-week follow-up. A single observer performed all echocardiographic analyses, scoring 16 predefined segments of the left ventricle (LV) as either normal or hyperkinetic (1 point), hypokinetic (2 points), akinetic (3 points), or dyskinetic (4 points) using a digital ultrasonic device (Vivid I or Vivid 7 with Echopac software (GE Vingmed Ultrasound, Horten, Norway). WMSI is calculated as the sum of scores divided by number of assessed segments, yielding a score = 1 for a normally functioning LV, and >1 in the case of LV dysfunction. Upon study inclusion, patients were required to have decreased LV wall motion in at least 3 of 16 segments. Left ventricular ejection fraction (LVEF) was assessed using Simpson’s biplane method or by visual approximation. Global longitudinal strain (GLS) measurements were derived from speckle tracking echocardiography images of the myocardium in three apical views, with automatic calculations by the aforementioned software. Analysis required optimal tracking in at least 14 segments and absence of AF.

Infarct size was quantified using 99 m-tetrofosmin ECG-gated myocardial single photon emission computed tomography (SPECT), expressing the perfusion defect as percentage of LV mass.

### Laboratory methods

Venepuncture was performed at baseline on study inclusion (median 22 hours after PCI), and subsequently on days 1, 2 5, and 42 (6-week follow-up). Plasma was collected using EDTA and citrated Vacutainer^®^ tubes, and then kept on ice until centrifugation within 30 min at 3000*g* and 4°C for 20 min. Serum was centrifuged at 2500*g* for 10 min within an hour of sampling. Samples were ultimately stored at -80°C pending analysis.

Plasma (EDTA) levels of double-stranded DNA (dsDNA) were quantified using a fluorescent nucleic acid stain, Quant-iT PicoGreen^®^ (Invitrogen Ltd., Paisley, UK) and a fluorimeter (Fluoroskan Ascent^®^, Thermo Fisher Scientific Oy, Vantaa, Finland) with an inter-assay coefficient of variation (CV) of 8.2%. Serum levels of myeloperoxidase-DNA (MPO-DNA) complexes were assessed using an enzyme-linked immunosorbent assay (ELISA) technique previously described by Kessenbrock et al. [13]. Briefly, the capture antibody anti-MPO (AbD Serotec, Hercules, CA, USA) was coated to plates and incubated overnight at 4°C. After blocking with BSA, patient serum and a peroxidase-labelled anti-DNA antibody (Cell Death Detection kit, Roche Diagnostics GmbH, Mannheim, Germany) were added. Finally, a peroxidase substrate was added and absorbance measured, expressed as optical density (OD) units with an inter-assay CV of 11.8%. Serum levels of citrullinated histone 3 (CitH3) were measured using a commercial sandwich ELISA kit (Cayman Chemical, Ann Arbor, MI, USA) with an inter-assay CV of 7.3%.

Circulating levels of IL-8 were quantified in serum using a commercial ELISA (R&D Systems, Abingdon, UK) with an inter-assay CV of 7.6%. Peak cardiac troponin T (TnT) was measured in serum using a commercial immunoassay (third generation cTroponin T, Elecys 2010, Roche, Mannheim, Germany).

### Statistical analysis

Data are presented as mean (±SD), median (25^th^, 75^th^ percentile), or proportions (%) as appropriate. The distribution of data was judged based on skewness, kurtosis and Q-Q plots (S1 File). As the measured NETs components were mostly skewly distributed, Spearman’s rho (*r*_*s*_) was used to perform correlation analyses, and 95% confidence intervals (CI) for rho were calculated using Fisher’s Z transformation. Continuous data were compared by use of the unpaired two-sample Student *t*-test or Mann-Whitney U test depending on data distribution. The Friedman test was performed to assess differences in levels of the NETs components across repeated samples, whereas Wilcoxon signed-rank tests were used to assess differences in the levels of the markers pairwise compared to baseline levels. Area under the curve (AUC) from baseline to day 5 was approximated for each NETs marker and IL-8 using the trapezoidal rule (*n* = 53 due to missing samples day 2). Longitudinal mixed model regression analysis was performed to assess the effects of NETs levels on myocardial function, testing for potential confounders (age, sex, smoking, Killip class, baseline creatinine level, baseline leukocyte count, and peak TnT) (S1 File). The level of statistical significance was set to *p* ≤ 0.05, and analyses were performed using IBM^®^SPSS^®^ Statistics software version 25 or R version 4.0.2 [14].

## Results

### Baseline characteristics

The patients had a mean age of 65 years and approximately 30% were women (Table 1). Only one patient had a history of chronic heart failure. With the exception of MPO-DNA levels on day 2, no significant differences in circulating NETs components were observed between groups receiving levosimendan or placebo. For this reason, and taking into account the small sample size, no further analyses were performed with respect to intervention groups. Characteristics according to above- and below-median levels of the NETs components are displayed in S1 Table.

**Table 1:** Baseline characteristics of the study population.

	Total study population (*n* = 61)	
Age, years, mean (range)	65	(34–90)
Female sex	18	(29.5)
Current smoking	22	(37.3)
Hypertension	21	(34.4)
Dyslipidaemia	13	(21.3)
Diabetes mellitus	6	(9.8)
Previous MI	11	(18.0)
Cardiogenic shock	9	(14.8)
Multiple vessel disease on angiography	31	(50.8)
IRA—Left anterior descending artery	46	(75.4)
•Left circumflex artery	8	(13.1)
•Right coronary artery	3	(4.9)
•Left main stem	4	(6.6)
WMSI at baseline	1.98	(± 0.23)
GLS at baseline	-9.07	(± 2.17)
LVEF at baseline, %	41.2	(± 8.5)
NT-proBNP, ng/L	434	(264, 779)
Peak TnT, ng/L	12 915	(7811, 17523)
Total leukocyte count, x10^9^/L	12.7	(10.2, 15.8)
Creatinine, μmol/L	82	(68, 97)
Systolic blood pressure on admission	104	(± 16.3)
Diastolic blood pressure on admission	65	(± 9.8)
Hours from PCI to study infusion/baseline	22	(14, 29)

Values presented as mean (±SD) or (range), median (25^th^, 75^th^ percentiles) or numbers (%) as appropriate. *n*: Number of cases.

MI: Myocardial infarction.

IRA: Infarct-related artery.

WMSI: Wall motion score index.

GLS: Global longitudinal strain.

LVEF: Left ventricular ejection fraction.

NT-proBNP: N-terminal pro-brain natriuretic peptide.

TnT: Cardiac troponin T.

PCI: Percutaneous coronary intervention.

### Temporal profile of NETs components

**Fig 1:**
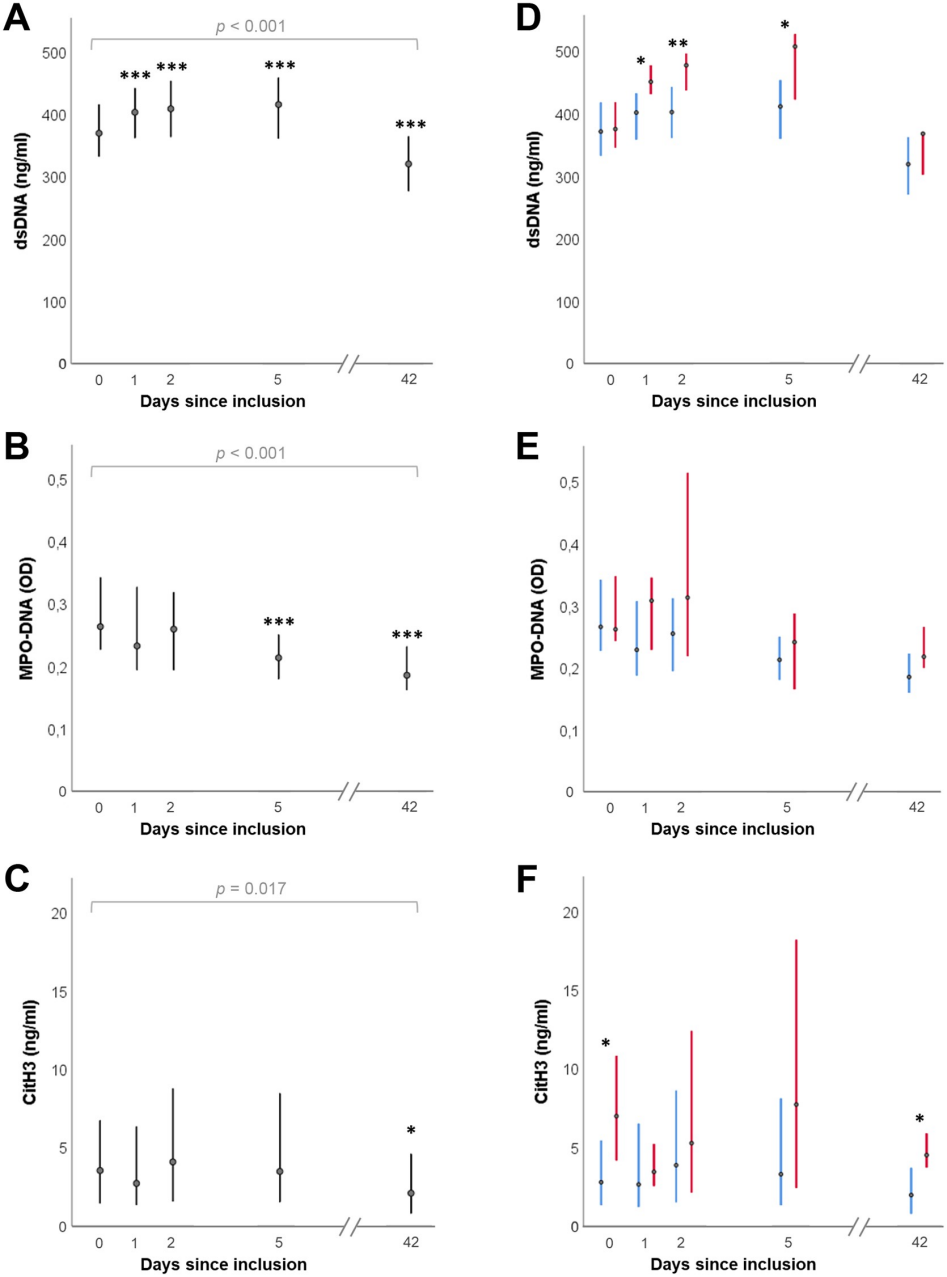
Temporal profile of the three NETs components (A-C), also stratified according to cardiogenic shock at inclusion (D-F). Points represent median values, and the lines represent interquartile ranges. Red and blue lines indicate presence and absence of cardiogenic shock. P-values in A-C refer to Friedman tests assessing differences in levels of the NETs components across repeated samples. Asterisks in A-C represent significant differences from baseline levels based on Wilcoxon signed-rank tests, and asterisks in D-F refer to Mann-Whitney U tests comparing groups with or without cardiogenic shock (*: p ≤ 0.05, **: p ≤ 0.01, ***: p ≤ 0.001). The number of cases (*n*) at the five consecutive time points were in A: 61, 60, 53, 59, 53 and for B-C: 61, 60, 52, 59, 51. Of these cases, the number with cardiogenic shock were in D: 9, 9, 6, 8, 6 and for E-F: 9, 9, 6, 8, 5.

There was a significant difference in the levels of all three NETs components across the five sampling time points from baseline to six weeks (day 42), assessed using Friedman tests (*p* ≤ 0.017 for all) (Fig 1A–1C). dsDNA levels increased significantly from baseline to day 1, day 2, and day 5 when levels seemed to peak, whereas MPO-DNA decreased significantly from baseline to day 5 (*p* < 0.001). No significant changes were observed in CitH3 levels during the first five days. When comparing levels of the NETs components at baseline through day 5 to the levels at six weeks, both dsDNA and MPO-DNA were significantly higher at all previous time points (p < 0.001 for all for dsDNA, and p < 0.05 for all for MPO-DNA). CitH3 was only significantly higher at baseline, day 2 and day 5 (p < 0.04) and just missed significance on day 1 (p = 0.06).

The AUCs for the three NETs components, expressing total “burden” over the first five days, hereafter referred to as dsDNA_AUC_, MPO-DNA_AUC_ and CitH3_AUC_, were 2000 ng/ml∙days (1824, 2209), 1.29 OD∙days (1.05, 1.45), and 26.4 ng/ml∙days (11.0, 39.2), respectively.

Stratifying patients according to the presence (*n* = 9) or absence (*n* = 52) of cardiogenic shock at baseline, there were no significant between-group differences in dsDNA levels at baseline or at 6-week follow-up, but dsDNA was significantly higher in the group with shock as compared to patients without shock on day 1, 2, and 5 (Fig 1D). MPO-DNA levels did not differ significantly between the groups at any time point, whereas the patients with shock had significantly higher CitH3 levels at baseline and at the 6-week follow-up compared to patients without shock (Fig 1E and 1F). Patients with cardiogenic shock (*n* = 6), as compared to those without, had significantly greater dsDNA_AUC_ (2266 ng/ml∙days (2117, 2547) vs 1982 (1792, 2122), *p* = 0.02) and CitH3_AUC_ (49.8 ng/ml∙days (29.3, 51.6) vs. 24.3 (9.9, 36.6), *p* = 0.02), but no significant difference was observed between the two groups for MPO-DNA_AUC_ (*p* = 0.10).

### NETs components and indices of myocardial function

**Fig 2:**
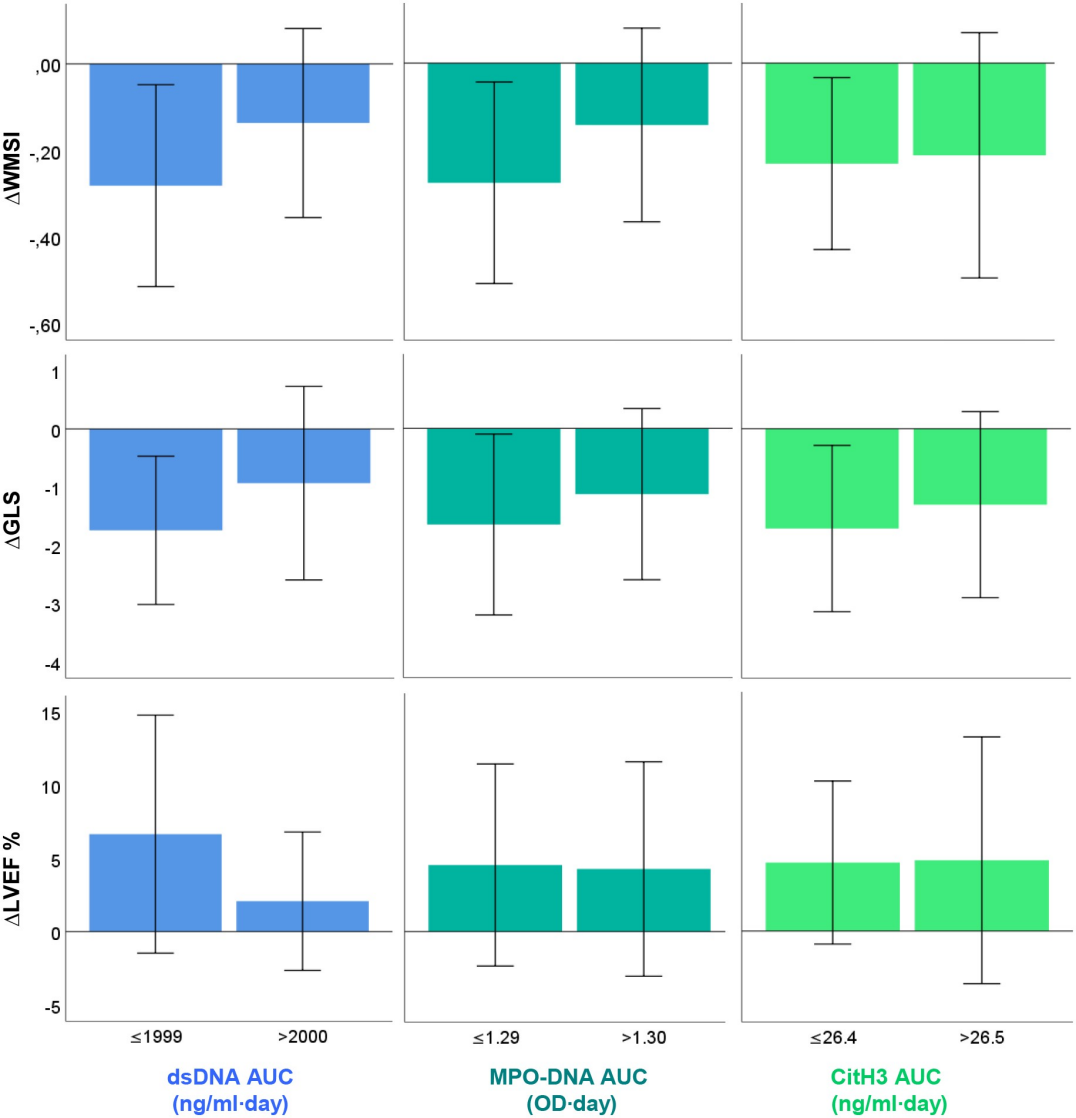
Mean change (Δ) in WMSI, GLS, and LVEF from baseline to day 5, according to below- or above-median levels of dsDNA_AUC_, MPO-DNA_AUC_, and CitH3_AUC_. Error bars indicate one standard deviation from the mean. The number of cases (*n*) analysed for WMSI, GLS and LVEF were 52, 44, and 52, respectively.

dsDNA_AUC_ and MPO-DNA_AUC_ were significantly correlated with change in WMSI from baseline to day 5 (*r*_*s*_ = 0.28 for both, *p* = 0.04–0.05, 95% CI 0.01 to 0.51 and 0.01 to 0.52 respectively) (S2 Table). No significant correlations to change in WMSI were observed for CitH3_AUC_. There were no significant correlations between NETs AUCs and change in GLS or LVEF during the first five days (S2 Table). In order to visualise potential effects with respect to changes in myocardial function, NETs AUCs were dichotomised at median levels as shown in Fig 2.

To investigate potential effects of NETs components on changes in myocardial function as illustrated in Fig 2, longitudinal mixed model regression analysis was performed (S1 File). Neither crude models nor models adjusting for potential confounders could identify a significant main effect of the NETs components on any of the echocardiographic parameters. Nor were there any significant effects when including an interaction between the NETs components and time.

### NETs components and interleukin 8

**Fig 3:**
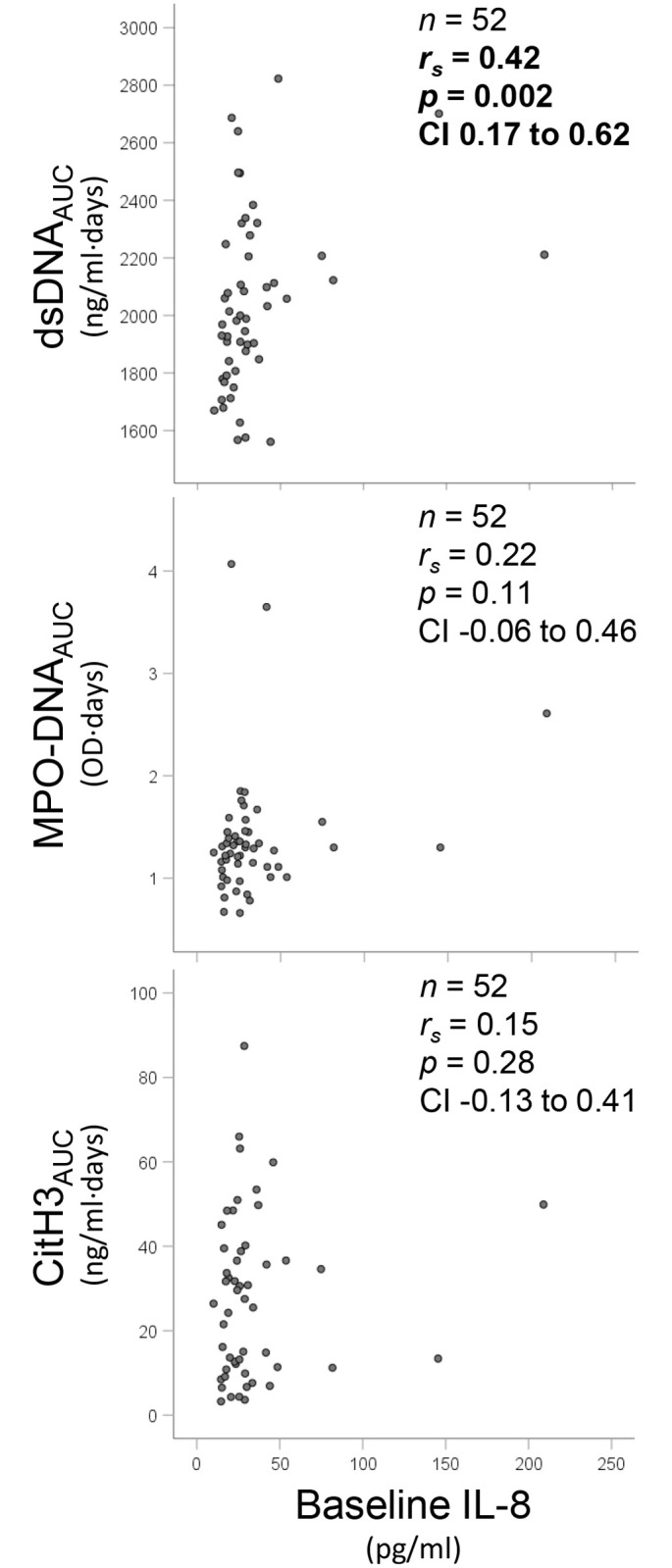
Correlation matrix between the “burden” (AUC) of the three NETs components and IL-8 levels at baseline. r_s_ refers to Spearman’s rho with corresponding p-values. For each correlation *n* refers to the number of case analysed, and CI is the 95% confidence interval for Spearman’s rho, calculated by Fisher Z transformation.

Correlations between NETs levels and IL-8 at corresponding time points are shown in Table 2, while correlations between NETs AUCs and IL-8 at baseline are presented in Fig 3. dsDNA_AUC_ was significantly correlated with baseline IL-8 (*r*_*s*_ = 0.42, *p* = 0.002, 95% CI 0.17 to 0.62). However when investigating potential mediating effects of the NETs components on IL-8 effects in the aforementioned mixed model regression analysis, no significant effects were observed.

**Table 2:** Correlations between NETs markers and IL-8 levels at corresponding sampling time points.

		IL-8				
		BL	Day 1	Day 2	Day 5	6 w
dsDNA	*n*	60	59	52	59	53
	*r*_*s*_	0.14	**0.26**	**0.30**	**0.53**	**0.39**
	*p*	0.28	**0.05**	**0.03**	**<0.001**	**0.004**
	*95% CI*	-0.12 to 0.38	**0.005 to 0.48**	**0.03 to 0.53**	**0.32 to 0.69**	**0.13 to 0.60**
MPO-DNA	*n*	60	59	52	59	51
	*r*_*s*_	0.10	0.19	**0.35**	**0.27**	0.09
	*p*	0.43	0.16	**0.01**	**0.04**	0.53
	*95% CI*	-0.15 to 0.35	-0.07 to 0.42	**0.08 to 0.56**	**0.02 to 0.49**	-0.191 to 0.36
CitH3	*n*	60	59	52	59	51
	*r*_*s*_	-0.08	0.02	0.08	**0.34**	**0.31**
	*p*	0.54	0.86	0.57	**0.01**	**0.03**
	*95% CI*	-0.33 to 0.18	-0.24 to 0.27	-0.20 to 0.35	**0.09 to 0.54**	**0.04 to 0.54**

*n*: Number of cases.

*r*_*s*_: Spearman’s rho.

CI: Confidence interval for Spearman’s rho calculated using the Fisher Z transformation.

dsDNA: Double-stranded DNA.

MPO-DNA: Myeloperoxidase-DNA complexes.

CitH3: Citrullinated histone 3.

IL-8: Interleukin 8.

BL: Baseline.

6 w: Six weeks.

### NETs components and indices of myocardial injury

Myocardial infarct size, as determined by SPECT after 6 weeks, did not correlate significantly with levels of the NETs components at any time point, nor with NETs AUCs (S3 Table). dsDNA_AUC_ correlated significantly with peak TnT levels (*r*_*s*_ = 0.44, *p* = 0.001, n = 53, 95% CI 0.20 to 0.64), whereas MPO-DNA_AUC_ and CitH3_AUC_ did not. Neither peak TnT nor SPECT-determined infarct size differed significantly between cardiogenic shock groups.

## Discussion

In this study of 61 patients with AHF complicating a PCI-treated STEMI, we could not confirm that NETs components play a role in myocardial recovery after STEMI. The association with IL-8, although weak, may support IL-8 to be a potential NETs inducer in this setting. Although no assertions can be made with respect to causality, to the best of our knowledge, this is the first report on the temporal profile of circulating NETs components in AHF complicating STEMI.

Although the total burden of dsDNA and MPO-DNA were associated with change in WMSI from baseline to day 5, longitudinal mixed model analysis showed neutral effects with respect to NETs and myocardial recovery. Although not specifically examining AHF patients, NETs burden has previously been linked to infarct size and left ventricular function [10,15]. Neutrophils are known to be involved in plugging of the microvasculature during myocardial IR-injury [16], and NETs may in this way contribute to the loss of viable myocardium that occurs even though coronary artery patency has been achieved by PCI. By use of murine models of myocardial IR-injury, several groups have reported that NETs destruction by DNase seems to reduce IR-injury and adverse left ventricular remodelling, presumably by abrogating microthrombosis and the cytotoxic effects of histones [17–19]. In line with these observations, we have recently observed high levels of circulating dsDNA to be associated with microvascular obstruction in patients with PCI-treated STEMI [11]. The proposed adverse effects of NETs structures on the myocardium could also be the result of proinflammatory activity, as NETs have been shown to stimulate cytokine release from other immune cells [20]. A higher NETs burden might reflect a greater intensity of the inflammatory response after infarction, which in turn is thought to disrupt myocardial healing [4]. The pathophysiologic mechanisms linking NETs to myocardial function are far from adequately understood, and unfortunately our data did not support a clear involvement of NETs components in myocardial recovery after STEMI.

Despite some significant correlations between WMSI and NETs burden, the findings for GLS and LVEF were inconsistent. The observations with respect to changes in LVEF may be obscured by the compensation of non-infarcted myocardium, supported by evidence from one cohort with myocardial infarction in which most had LVEF within the normal range, whilst 70% had impaired GLS [21]. Despite moderate correlations between the different echocardiographic measures of change (S4 Table), our inconsistent observations nonetheless highlight the challenge of interpreting myocardial function parameters in the setting of AHF complicating STEMI, and the need for further research.

NETs burden as quantified by dsDNA and CitH3 was significantly higher amongst patients with cardiogenic shock, but with no association to final infarct size, supporting the observation that NETs burden may be partly related to heart failure with increas0065d filling pressure and low cardiac output, and not only myocardial necrosis. Increased NETosis in cardiogenic shock may suggest that NETs components are released as a result of systemic hypoperfusion and organ hypoxia, with NETosis potentially being triggered on a broader scale. In line with this, dsDNA, MPO-DNA and histones have all been reported to be elevated in patients with septic shock [22,23], leading one to speculate that cellular injury processes outside the coronary circulation or non-specific to neutrophils could be important.

An association between IL-8 and NETosis was only corroborated by the correlation between dsDNA and IL-8 at baseline. In this cohort, we have previously shown that IL-8 levels were significantly higher at baseline than at all later points [6], seemingly peaking prior to the peaks of the NETs components. Although only conjectural, this is in line with *in vitro* data showing that IL-8 stimulates NETs release [7], though no data on circulating markers have to our knowledge previously been reported. The lack of correlations between IL-8 and MPO-DNA and CitH3 could hypothetically indicate that IL-8 is involved in other cellular processes resulting in the extrusion of nuclear material, not limited to NETs release.

With respect to the neutral effects of NETs components on myocardial recovery, it might be discussed whether some of the neutrophil components also act to *limit* the inflammatory response. For instance, NETs could perhaps restrict injury to a smaller area, trigger compensation of the remote non-infarcted myocardium, or initiate repair, however such causal inferences cannot be drawn from the present study. The crucial role of neutrophils in the conversion of macrophages to a reparative phenotype, as well as the timely development of a fibrotic scar, is evident from studies in mice showing that neutrophil depletion leads to poorer myocardial function and the development of AHF [24]. Recent data from a murine model of MI indicates that NETs may promote resolution of inflammation [25] and yet another study showed that NETs likely influence the conversion of monocytes to scar-generating fibrocytes [15]. Other beneficial effects of neutrophils could mitigate the potential detrimental effects of NETs, complicating interpretation of our results. Finally, although levosimendan is proposed to have pleiotropic anti-inflammatory effects, potentially reducing neutrophil activation and degranulation [26], we could not find any significant difference between treatment groups in this study. We have previously shown that levosimendan did not affect circulating markers of inflammation in this cohort [6].

### Limitations

Limitations of this study include sample size, potential impact of outliers, and the number of analyses performed, although the study is exploratory and observational in nature, precluding conclusions with respect to causality. Whether dsDNA is a nonspecific marker of cellular injury or a marker of NETosis cannot be determined from our data, nor can we dissect whether our observations are related primarily to MI or AHF. Moreover, we can only speculate as to possible reasons for the inconsistent observations between the different NETs components, potentially due to differential release of the individual components, differing half-life in the circulation, or methodological issues. The primary endpoint (change in WMSI from baseline to day 5) was in part chosen based on the pharmacokinetic properties of levosimendan, thus limiting the study hypothesis to neutrophil effects in the acute and subacute phase. Finally, concurrent conditions such as infections may have affected neutrophil activity and general inflammation.

## Conclusions

Our results provide novel knowledge of how levels of circulating NETs components evolve in patients with large myocardial infarctions and symptomatic heart failure. Despite some correlations between NETs components and myocardial recovery after STEMI, and the observation that IL-8 might be involved in enhancing NETs formation in AHF, no significant cause-effect relationships could be shown. The mechanistic links and potential clinical utility of NETs components as biomarkers in AHF require further investigation.

## Supporting information

S1 Fig LEAF study flow chart. (DOCX)

S1 Table Selected baseline characteristics of the study population according to above- or below-median levels of the area under the curve (AUC) of the three NETs components. (DOCX)

S2 Table Correlations between area under the curve (AUC) of the three NETs markers, and change (Δ) in indices of myocardial function from baseline to day 5. (DOCX)

S3 Table Correlations between myocardial infarct size as determined by SPECT after 6 weeks and the three NETs markers, at each time point and total “burden” or area under the curve (AUC). (DOCX)

S4 Table Correlations between echocardiographic measures of change in myocardial function from baseline to day 5. (DOCX)

S1 File Appendix of statistical analyses. (PDF)

S2 File Minimal anonymized dataset. (XLSX)

## References

[1] Desta L, Jernberg T, Lofman I, Hofman-Bang C, Hagerman I, Spaak J, et al Incidence, temporal trends, and prognostic impact of heart failure complicating acute myocardial infarction. The SWEDEHEART Registry (Swedish Web-System for Enhancement and Development of Evidence-Based Care in Heart Disease Evaluated According to Recommended Therapies): a study of 199,851 patients admitted with index acute myocardial infarctions, 1996 to 2008. *JACC Heart Fail*. 2015;3(3):234–242. 10.1016/j.jchf.2014.10.007 25742760

[2] Bartekova M, Radosinska J, Jelemensky M, Dhalla NS. Role of cytokines and inflammation in heart function during health and disease. *Heart Fail Rev*. 2018;23(5):733–758. 10.1007/s10741-018-9716-x 29862462

[3] Ong SB, Hernandez-Resendiz S, Crespo-Avilan GE, Mukhametshina RT, Kwek XY, Cabrera-Fuentes HA, et al Inflammation following acute myocardial infarction: Multiple players, dynamic roles, and novel therapeutic opportunities. *Pharmacol Ther*. 2018;186:73–87. 10.1016/j.pharmthera.2018.01.001 29330085 PMC5981007

[4] Westman PC, Lipinski MJ, Luger D, Waksman R, Bonow RO, Wu E, et al Inflammation as a Driver of Adverse Left Ventricular Remodeling After Acute Myocardial Infarction. *J Am Coll Cardiol*. 2016;67(17):2050–2060. 10.1016/j.jacc.2016.01.073 27126533

[5] Ma Y, Yabluchanskiy A, Lindsey ML. Neutrophil roles in left ventricular remodeling following myocardial infarction. *Fibrogenesis & tissue repair*. 2013;6(1):11–11. 10.1186/1755-1536-6-11 23731794 PMC3681584

[6] Husebye T, Eritsland J, Arnesen H, Bjornerheim R, Mangschau A, Seljeflot I, et al Association of interleukin 8 and myocardial recovery in patients with ST-elevation myocardial infarction complicated by acute heart failure. *PLoS One*. 2014;9(11):e112359 10.1371/journal.pone.0112359 25390695 PMC4229310

[7] Brinkmann V, Reichard U, Goosmann C, Fauler B, Uhlemann Y, Weiss DS, et al Neutrophil extracellular traps kill bacteria. *Science*. 2004;303(5663):1532–1535. 10.1126/science.1092385 15001782

[8] Döring Y, Soehnlein O, Weber C. Neutrophil Extracellular Traps in Atherosclerosis and Atherothrombosis. *Circ Res*. 2017;120(4):736–743. 10.1161/CIRCRESAHA.116.309692 28209798

[9] Sorvillo N, Cherpokova D, Martinod K, Wagner DD. Extracellular DNA NET-Works With Dire Consequences for Health. *Circ Res*. 2019;125(4):470–488. 10.1161/CIRCRESAHA.119.314581 31518165 PMC6746252

[10] Mangold A, Alias S, Scherz T, Hofbauer T, Jakowitsch J, Panzenbock A, et al Coronary neutrophil extracellular trap burden and deoxyribonuclease activity in ST-elevation acute coronary syndrome are predictors of ST-segment resolution and infarct size. *Circ Res*. 2015;116(7):1182–1192. 10.1161/CIRCRESAHA.116.304944 25547404

[11] Helseth R, Shetelig C, Andersen GO, Langseth MS, Limalanathan S, Opstad TB, et al Neutrophil Extracellular Trap Components Associate with Infarct Size, Ventricular Function, and Clinical Outcome in STEMI. *Mediators Inflamm*. 2019;2019:7816491 10.1155/2019/7816491 31772506 PMC6854936

[12] Husebye T, Eritsland J, Muller C, Sandvik L, Arnesen H, Seljeflot I, et al Levosimendan in acute heart failure following primary percutaneous coronary intervention-treated acute ST-elevation myocardial infarction. Results from the LEAF trial: a randomized, placebo-controlled study. *Eur J Heart Fail*. 2013;15(5):565–572. 10.1093/eurjhf/hfs215 23288914

[13] Kessenbrock K, Krumbholz M, Schönermarck U, Back W, Gross WL, Werb Z, et al Netting neutrophils in autoimmune small-vessel vasculitis. *Nat Med*. 2009;15(6):623–625. 10.1038/nm.1959 19448636 PMC2760083

[14] R Core Team. R: A language and environment for statistical computing. Vienna, Austria: R Foundation for Statistical Computing; 2020.

[15] Hofbauer TM, Mangold A, Scherz T, Seidl V, Panzenbock A, Ondracek AS, et al Neutrophil extracellular traps and fibrocytes in ST-segment elevation myocardial infarction. *Basic Res Cardiol*. 2019;114(5):33 10.1007/s00395-019-0740-3 31312919 PMC6647191

[16] Vinten-Johansen J. Involvement of neutrophils in the pathogenesis of lethal myocardial reperfusion injury. *Cardiovasc Res*. 2004;61(3):481–497. 10.1016/j.cardiores.2003.10.011 14962479

[17] Vogel B, Shinagawa H, Hofmann U, Ertl G, Frantz S. Acute DNase1 treatment improves left ventricular remodeling after myocardial infarction by disruption of free chromatin. *Basic Res Cardiol*. 2015;110(2):15 10.1007/s00395-015-0472-y 25702039

[18] Ge L, Zhou X, Ji WJ, Lu RY, Zhang Y, Zhang YD, et al Neutrophil extracellular traps in ischemia-reperfusion injury-induced myocardial no-reflow: therapeutic potential of DNase-based reperfusion strategy. *Am J Physiol Heart Circ Physiol*. 2015;308(5):H500–509. 10.1152/ajpheart.00381.2014 25527775

[19] Savchenko AS, Borissoff JI, Martinod K, De Meyer SF, Gallant M, Erpenbeck L, et al VWF-mediated leukocyte recruitment with chromatin decondensation by PAD4 increases myocardial ischemia/reperfusion injury in mice. *Blood*. 2014;123(1):141–148. 10.1182/blood-2013-07-514992 24200682 PMC3879903

[20] Warnatsch A, Ioannou M, Wang Q, Papayannopoulos V. Inflammation. Neutrophil extracellular traps license macrophages for cytokine production in atherosclerosis. *Science*. 2015;349(6245):316–320. 10.1126/science.aaa8064 26185250 PMC4854322

[21] Baron T, Christersson C, Hjorthen G, Hedin EM, Flachskampf FA. Changes in global longitudinal strain and left ventricular ejection fraction during the first year after myocardial infarction: results from a large consecutive cohort. *Eur Heart J Cardiovasc Imaging*. 2018;19(10):1165–1173. 10.1093/ehjci/jex260 29145641

[22] Maruchi Y, Tsuda M, Mori H, Takenaka N, Gocho T, Huq MA, et al Plasma myeloperoxidase-conjugated DNA level predicts outcomes and organ dysfunction in patients with septic shock. *Crit Care*. 2018;22(1):176 10.1186/s13054-018-2109-7 30005596 PMC6045839

[23] Stiel L, Meziani F, Helms J. Neutrophil activation during septic shock. *Shock*. 2018;29:29 10.1097/SHK.0000000000000980 28858142

[24] Horckmans M, Ring L, Duchene J, Santovito D, Schloss MJ, Drechsler M, et al Neutrophils orchestrate post-myocardial infarction healing by polarizing macrophages towards a reparative phenotype. *Eur Heart J*. 2017;38(3):187–197. 10.1093/eurheartj/ehw002 28158426

[25] Eghbalzadeh K, Georgi L, Louis T, Zhao H, Keser U, Weber C, et al Compromised Anti-inflammatory Action of Neutrophil Extracellular Traps in PAD4-Deficient Mice Contributes to Aggravated Acute Inflammation After Myocardial Infarction. *Front Immunol*. 2019;10:2313 10.3389/fimmu.2019.02313 31632398 PMC6779806

[26] Adam M, Meyer S, Knors H, Klinke A, Radunski UK, Rudolph TK, et al Levosimendan displays anti-inflammatory effects and decreases MPO bioavailability in patients with severe heart failure. *Sci Rep*. 2015;5:9704 10.1038/srep09704 25867530 PMC4394753

