# Supplementary figures and images for: Neutrophil extracellular trap components and myocardial recovery in post-ischemic acute heart failure
Source: PLoS One. 2020 Oct 29;15(10):e0241333. doi: 10.1371/journal.pone.0241333 (PMC7595325; doi:10.1371/journal.pone.0241333)

## S1 Fig. LEAF study flow chart.
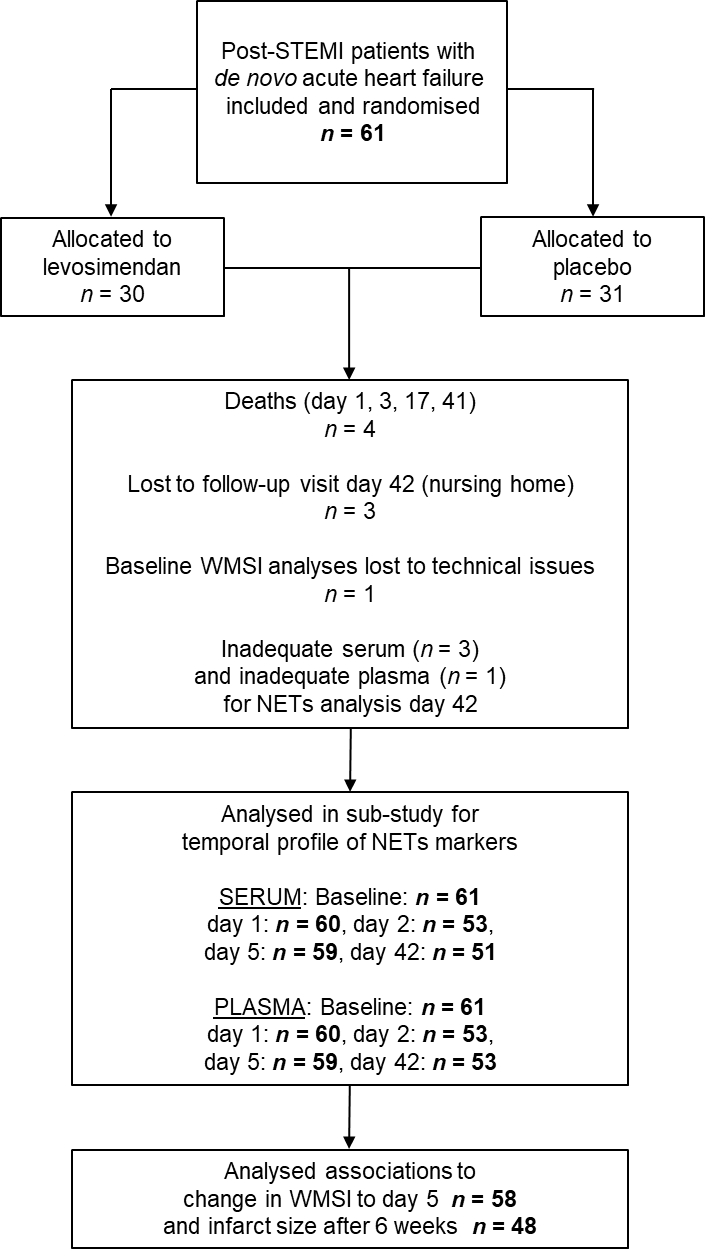

Supplement: S1 Fig — (DOCX) [file pone.0241333.s001.docx]
