# Supplementary material for: Neutrophil extracellular trap components and myocardial recovery in post-ischemic acute heart failure
Source: PLoS One. 2020 Oct 29;15(10):e0241333. doi: 10.1371/journal.pone.0241333 (PMC7595325; doi:10.1371/journal.pone.0241333)
Supplement: S1 Table — (DOCX) [file pone.0241333.s002.docx]

**S1 Table . Selected baseline characteristics of the study population according to above- or below-median levels of the area under the curve (AUC) of the three NETs components.**

|  | dsDNA_AUC_ | |  |  | MPO-DNA_AUC_ | |  | | CitH3_AUC_ | |  |  |
| --- | --- | --- | --- | --- | --- | --- | --- | --- | --- | --- | --- | --- |
|  | < median | ≥ median | *n*† | *p** | < median | ≥ median | *n*† | *p** | < median | ≥ median | *n*† | *p** |
| Age, mean (range) | 60.8 (34-83) | 67.5 (43-86) | 27/26 | **0.05** | 66.0 (43-86) | 62.0 (34-81) | 27/26 | 0.25 | 63.5 (34-86) | 64.3 (37-83) | 27/26 | 0.82 |
| Female sex | 11 (41) | 5 (19) | 27/26 | 0.09 | 9 (33) | 7 (27) | 27/26 | 0.61 | 8 (30) | 8 (31) | 27/26 | 0.93 |
| Current smoking | 7 (26) | 12 (48) | 27/25 | 0.10 | 7 (26) | 12 (48) | 27/25 | 0.10 | 6 (23) | 12 (46) | 26/26 | 0.08 |
| Hypertension | 8 (30) | 8 (31) | 27/26 | 0.93 | 7 (26) | 9 (35) | 27/26 | 0.49 | 5 (19) | 11 (42) | 27/26 | 0.06 |
| Diabetes mellitus | 2 (7) | 3 (12) | 27/26 | 0.61 | 2 (7) | 3 (12) | 27/26 | 0.61 | 4 (15) | 1 (4) | 27/26 | 0.17 |
| Cardiogenic shock | 1 (4) | 5 (19) | 27/26 | 0.08 | 1 (4) | 5 (19) | 27/26 | 0.08 | 1 (4) | 5 (19) | 27/26 | 0.08 |
| Multivessel disease  on angiography | 14 (52) | 12 (46) | 27/26 | 0.68 | 15 (56) | 11 (42) | 27/26 | 0.34 | 14 (52) | 12 (46) | 27/26 | 0.68 |
| IRA   - Left anterior descending  - Left circumflex artery  - Right coronary artery  - Left main stem | 21 (78) 4 (15) 0 (0) 2 (7) | 18 (69) 4 (15) 3 (12) 1 (4) | 27/26 | 0.32 | 20 (74) 3 (11) 1 (4) 3 (11) | 19 (73) 5 (19) 2 (8) 0 (0) | 27/26 | 0.28 | 19 (70) 4 (15) 2 (7) 2 (7) | 19 (73) 4 (15) 1 (4) 2 (8) | 27/26 | 0.96 |
| WMSI at baseline | 2.00 (±0.19) | 1.92 (±0.24) | 26/26 | 0.21 | 2.00 (±0.25) | 1.93 (±0.18) | 26/26 | 0.25 | 2.02 (±0.18) | 1.91 (±0.24) | 26/26 | 0.07 |
| GLS at baseline | -9.16 (±1.46) | -9.35 (±2.40) | 23/22 | 0.75 | -9.09 (±1.71) | -9.39 (±2.16) | 20/25 | 0.61 | -8.63 (±1.59) | -9.76 (±2.11) | 20/25 | **0.05** |
| LVEF at baseline, % | 42.9 (±9.3) | 43.1 (±7.2) | 26/26 | 0.93 | 43.3 (±8.8) | 42.7 (±7.8) | 26/26 | 0.78 | 40.7 (±7.7) | 45.2 (±8.2) | 26/26 | **0.05** |
| NT-proBNP, ng/L | 359 (222, 560) | 690 (296, 1472) | 27/26 | **0.02** | 560 (240, 922) | 386 (281, 638) | 27/26 | 0.59 | 498 (240, 922) | 355 (281, 680) | 27/26 | 0.52 |
| Peak TnT, ng/L | 11 440 (5670, 15870) | 15 155 (11309, 18265) | 27/26 | **0.008** | 11 828 (8066, 16000) | 14 326 (7923, 17848) | 27/26 | 0.56 | 13 597 (10570, 16746) | 13 322 (6608, 17825) | 27/26 | 0.65 |
| Total leukocyte count,  x10^9^/L | 13.3 (10.4, 15.3) | 12.7 (9.9, 16.3) | 27/25 | 0.85 | 12.7 (10.1, 15.8) | 14.3 (10.7, 16.5) | 27/25 | 0.33 | 12.7 (9.5, 15.8) | 14.3 (11.1, 16.3) | 27/25 | 0.35 |
| Creatinine, µmol/L | 78 (62, 90) | 83 (71, 125) | 27/26 | **0.05** | 81 (68, 93) | 80 (60, 98) | 27/26 | 0.59 | 81 (62, 97) | 80 (69, 98) | 27/26 | 0.81 |

Values presented as mean (±SD) or (range), median (25^th^, 75^th^ percentiles) or numbers (%) as appropriate.
* *p*-value of Mann-Whitney U, Student’s t, or Chi squared tests comparing groups with below- and above-median levels of the NETs AUCs.

† *n* cases (below-median AUC group / above-median AUC group) included in analysis

dsDNA: double-stranded DNA
MPO-DNA: myeloperoxidase-DNA complexes

CitH3: citrullinated histone 3

IRA: Infarct-related artery

WMSI: Wall motion score index

GLS: Global longitudinal strain

LVEF: Left ventricular ejection fraction

NT-proBNP: N-terminal pro-brain natriuretic peptide

TnT: cardiac troponin T
