# Supplementary material for: Neutrophil extracellular trap components and myocardial recovery in post-ischemic acute heart failure
Source: PLoS One. 2020 Oct 29;15(10):e0241333. doi: 10.1371/journal.pone.0241333 (PMC7595325; doi:10.1371/journal.pone.0241333)
Supplement: S2 Table — (DOCX) [file pone.0241333.s003.docx]

**S2 Table. Correlations between area under the curve (AUC) of the three NETs markers, and change (Δ) in indices of myocardial function from baseline to day 5.**

|  |  | dsDNA_AUC_ | MPO-DNA_AUC_ | CitH3_AUC_ |
| --- | --- | --- | --- | --- |
| ∆WMSI  (BL-Day5) | *n*  *r_s_*  *p*  *95% CI* | 52  **0.28**  **0.05 0.01 to 0.51** | 52 **0.28**  **0.04 0.01 to 0.52** | 52 0.01  0.94  -0.26 to 0.28 |
| ∆GLS  (BL-Day5) | *n*  *r_s_*  *p*  *95% CI* | 44 0.11  0.47 -0.19 to 0.39 | 44 0.14  0.38 -0.17 to 0.42 | 44 0.05  0.74  -0.25 to 0.34 |
| ∆ LVEF (BL-Day5) | *n*  *r_s_*  *p*  *95% CI* | 52 -0.25  0.08 -0.49 to 0.02 | 52  -0.05  0.75 -0.32 to 0.23 | 52  -0.01  0.92  -0.28 to 0.26 |

*n:* Number of cases
r_s_: Spearman’s rho.

CI: Confidence interval for Spearman’s rho calculated using the Fisher Z transformation.

dsDNA: double-stranded DNA

MPO-DNA: myeloperoxidase-DNA complexes

CitH3: citrullinated histone 3

AUC: area under the curve from baseline to day 5

BL: baseline

WMSI: wall motion score index

GLS: global longitudinal strain

LVEF: left ventricular ejection fraction
