# Supplementary material for: Neutrophil extracellular trap components and myocardial recovery in post-ischemic acute heart failure
Source: PLoS One. 2020 Oct 29;15(10):e0241333. doi: 10.1371/journal.pone.0241333 (PMC7595325; doi:10.1371/journal.pone.0241333)
Supplement: S3 Table — (DOCX) [file pone.0241333.s004.docx]

**S3 Table. Correlations between myocardial infarct size as determined by SPECT after 6 weeks and the three NETs markers, at each time point and total “burden” or area under the curve (AUC).**

|  |  |  | Infarct size  (% of left ventricle) |
| --- | --- | --- | --- |
| dsDNA | Baseline | *n*  *r_s_*  *p 95% CI* | 48  -0.15  0.30 -0.42 to 0.14 |
|  | Day 1 | *n*  *r_s_*  *p 95% CI* | 48  -0.07  0.64  -0.35 to 0.22 |
|  | Day 2 | *n*  *r_s_*  *p 95% CI* | 44  0.07  0.65  -0.23 to 0.36 |
|  | Day 5 | *n*  *r_s_*  *p 95% CI* | 48  -0.19  0.20  -0.45 to 0.10 |
|  | Day 42 | *n*  *r_s_*  *p 95% CI* | 47  -0.17  0.26 -0.43 to 0.13 |
|  | dsDNA_AUC_ | *n*  *r_s_*  *p 95% CI* | 44  -0.07  0.66 -0.36 to 0.23 |
| MPO-DNA | Baseline | *n*  *r_s_*  *p 95% CI* | 48  -0.04  0.78 -0.32 to 0.25 |
|  | Day 1 | *n*  *r_s_*  *p 95% CI* | 48  0.11  0.45 -0.18 to 0.38 |
|  | Day 2 | *n*  *r_s_*  *p 95% CI* | 44  -0.13  0.39 -0.41 to 0.17 |
|  | Day 5 | *n*  *r_s_*  *p 95% CI* | 48  0.04  0.77 -0.24 to 0.32 |
|  | Day 42 | *n*  *r_s_*  *p 95% CI* | 45  -0.20  0.19 -0.46 to 0.10 |
|  | MPO-DNA_AUC_ | *n*  *r_s_*  *p 95% CI* | 44  -0.05  0.73 -0.34 to 0.25 |
| CitH3 | Baseline | *n*  *r_s_*  *p 95% CI* | 48  -0.16  0.28 -0.42 to 0.13 |
|  | Day 1 | *n*  *r_s_*  *p 95% CI* | 48  -0.14  0.33 -0.41 to 0.15 |
|  | Day 2 | *n*  *r_s_*  *p 95% CI* | 44  0.04  0.79 -0.26 to 0.33 |
|  | Day 5 | *n*  *r_s_*  *p 95% CI* | 48  -0.06  0.66 -0.34 to 0.22 |
|  | Day 42 | *n*  *r_s_*  *p 95% CI* | 45  -0.02  0.88 -0.31 to 0.27 |
|  | CitH3_AUC_ | *n*  *r_s_*  *p 95% CI* | 45  -0.01  0.95 -0.30 to 0.29 |

*n:* number of cases

*r_s_*: Spearman’s rho.
CI: Confidence interval for Spearman’s rho calculated using the Fisher Z transformation.

dsDNA: double-stranded DNA

MPO-DNA: myeloperoxidase-DNA complexes

CitH3: citrullinated histone 3

AUC: area under the curve from baseline to day 5
