# Supplementary material for: Neutrophil extracellular trap components and myocardial recovery in post-ischemic acute heart failure
Source: PLoS One. 2020 Oct 29;15(10):e0241333. doi: 10.1371/journal.pone.0241333 (PMC7595325; doi:10.1371/journal.pone.0241333)
Supplement: S4 Table — (DOCX) [file pone.0241333.s005.docx]

**S4 Table. Correlations between echocardiographic measures of change in myocardial function from baseline to day 5.**

|  |  | ∆GLS  (BL-Day5) | ∆LVEF  (BL-Day5) |
| --- | --- | --- | --- |
| ∆WMSI  (BL-Day5) | *n*  *r*  *p*  *95% CI* | 48  0.55  <0.001 0.32 to 0.72 | 58  -0.54  <0.001 -0.70 to -0.33 |
| ∆GLS  (BL-Day5) | *n*  *r*  *p 95% CI* | - | 48  -0.39  0.006 -0.61 to -0.12 |

*n:* number of cases
 *r*: Pearson’s correlation coefficient.
CI: Confidence interval for Spearman’s rho calculated using the Fisher Z transformation.

BL: baseline

WMSI: wall motion score index

GLS: global longitudinal strain

LVEF: left ventricular ejection fraction
