# Supplementary material for: Neutrophil extracellular trap components and myocardial recovery in post-ischemic acute heart failure
Source: PLoS One. 2020 Oct 29;15(10):e0241333. doi: 10.1371/journal.pone.0241333 (PMC7595325; doi:10.1371/journal.pone.0241333)
Supplement: S1 File — (PDF) [file pone.0241333.s006.pdf]

S1 File: Appendix of statistical analyses

Neutrophil extracellular trap components associate with myocardial recovery in post-ischemic acute heart failure

Miriam Sjåstad Langseth [1, 2, 4*], Geir Øystein Andersen [1, 3], Trygve Husebye [3], Harald Arnesen [1, 2], Manuela Zucknick [5], Svein Solheim [1, 3], Jan Eritsland [3], Ingebjørg Seljeflot [1, 2, 3], Trine Baur Opstad [1, 2], Ragnhild Helseth [1, 3].

[1] Center for Clinical Heart Research, Oslo University Hospital Ullevål, Oslo, Norway

[2] Faculty of Medicine, University of Oslo, Oslo, Norway

[3] Department of Cardiology, Oslo University Hospital Ullevål, Oslo, Norway

[4] Department of Internal Medicine, Drammen Hospital, Vestre Viken HF, Drammen, Norway

5 Oslo Centre for Biostatistics and Epidemiology, Institute for Basic Medical Sciences, University of Oslo, Oslo, Norway

* Corresponding author

E-mail address: m.s.langseth@studmed.uio.no (MSL)

Assessing distributions of the variables

Variable	*n*	skewness	kurtosis	D*	*p**	W**	*p***	Normal Q-Q plot	Normal distribution?
**dsDNA$_{AUC}$**	53	0.66	0.12	0.09	0.20	0.96	0.08	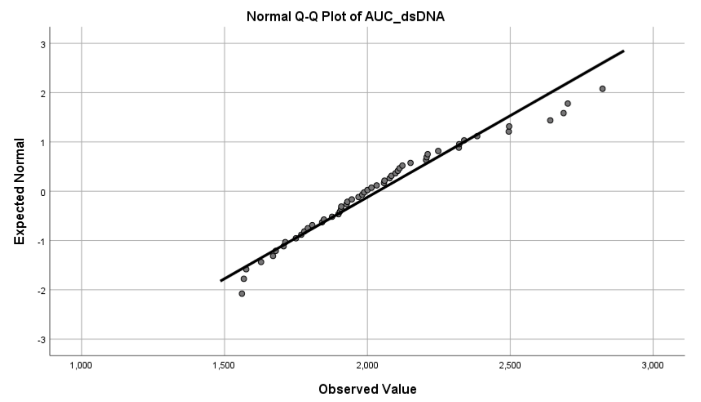	YES
**MPO-DNA$_{AUC}$**	53	2.93	10.59	0.23	<0.001	0.70	<0.001	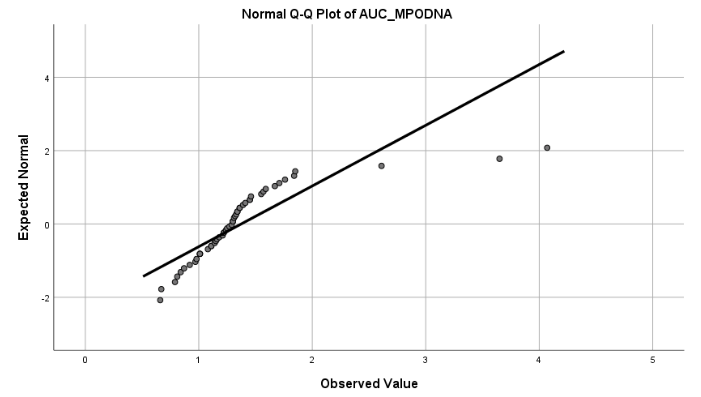	NO
**CitH3$_{AUC}$**	53	0.81	0.36	0.15	0.003	0.92	0.002	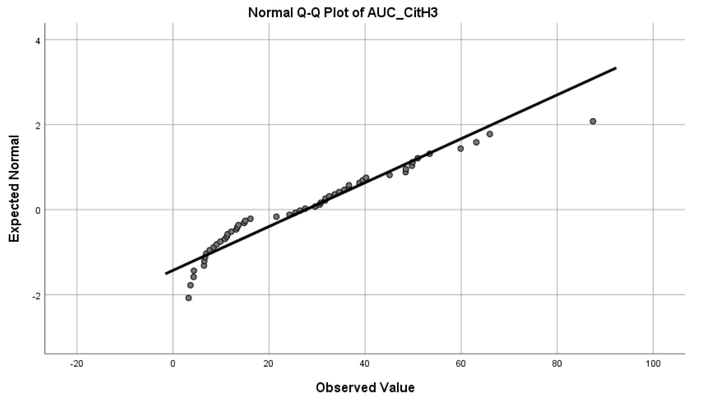	NO

**IL-8$_{AUC}$**	53	1.71	3.96	0.15	0.006	0.84	<0.001	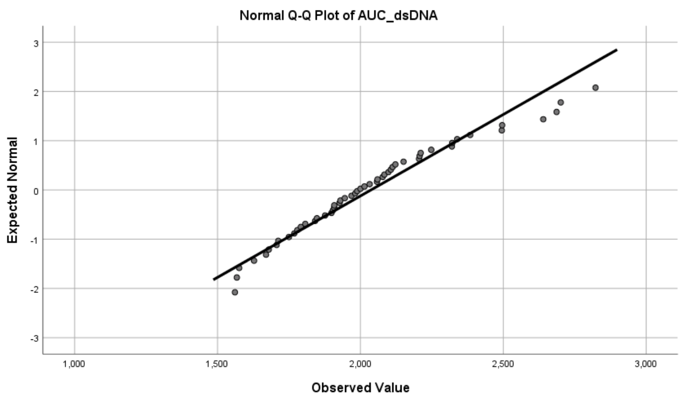	NO
**ΔWMSI BL-day5**	58	-0.02	0.64	0.09	0.20	0.98	0.49	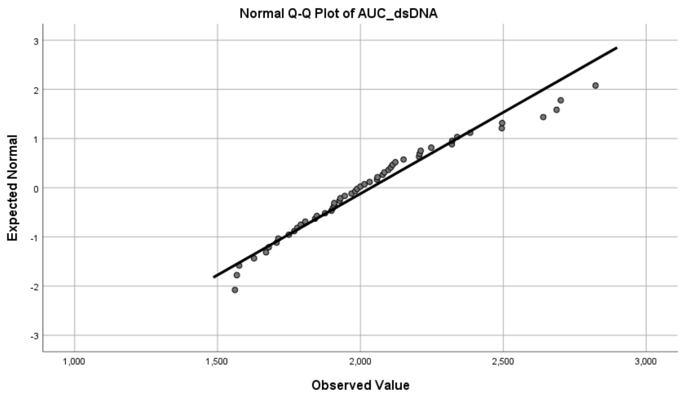	YES
**ΔGLS BL-day5**	48	0.24	0.01	0.07	0.20	0.98	0.57	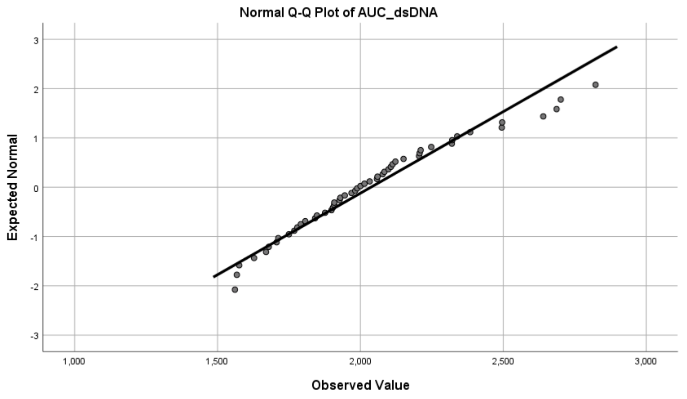	YES

**ΔEF BL-day5**	58	1.35	2.91	0.14	0.005	0.91	<0.001	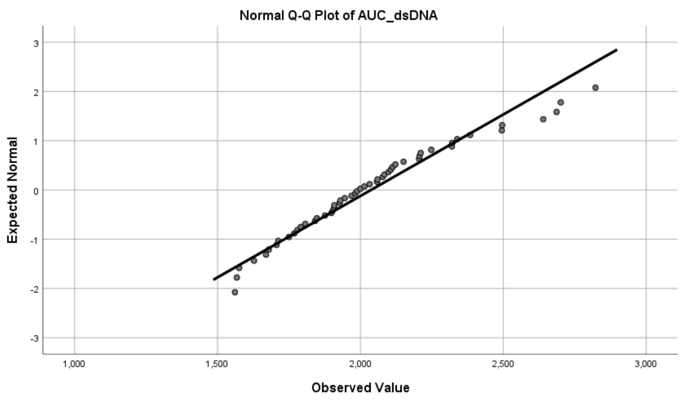	NO
**dsDNA BL**	61	0.46	0.46	0.08	0.20	0.98	0.51	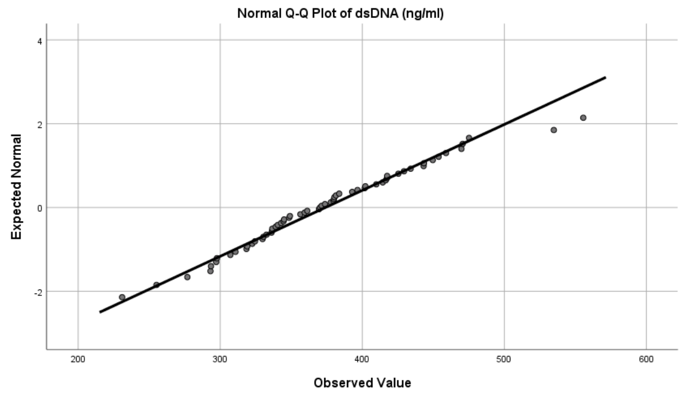	YES
**dsDNA day 1**	60	-0.10	2.80	0.10	0.20	0.95	0.02	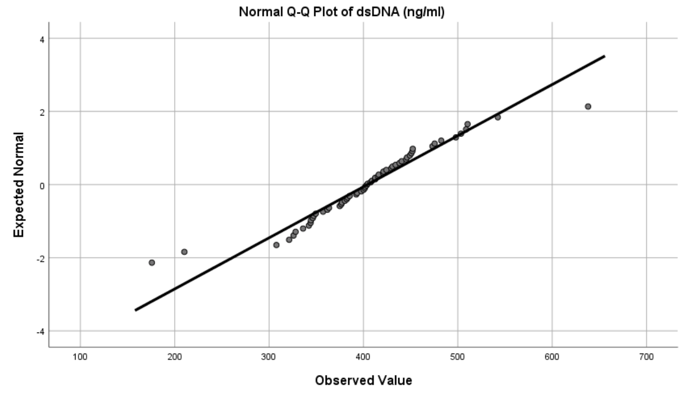	NO

**dsDNA day 2**	53	1.19	3.72	0.07	0.20	0.93	0.006	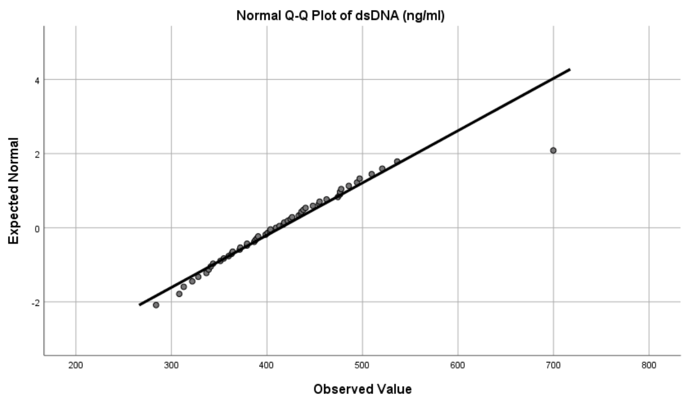	NO
**dsDNA day 5**	59	0.21	2.20	0.09	0.20	0.96	0.04	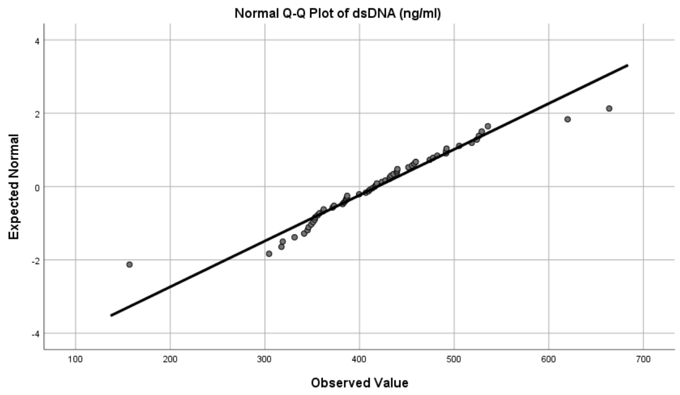	YES
**dsDNA day 42**	53	0.29	-0.89	0.09	0.20	0.96	0.05	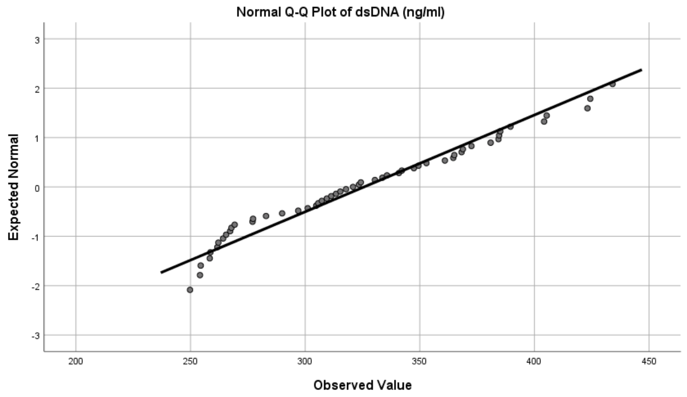	YES

**MPO-DNA BL**	61	2.91	10.20	0.20	<0.001	0.69	<0.001	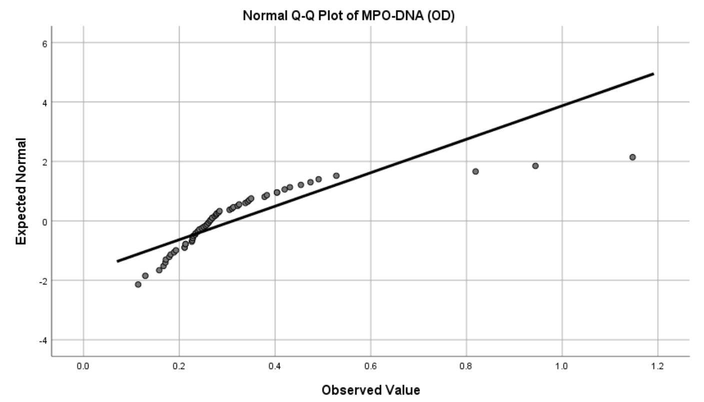	NO
**MPO-DNA day 1**	60	2.65	8.39	0.19	<0.001	0.72	<0.001	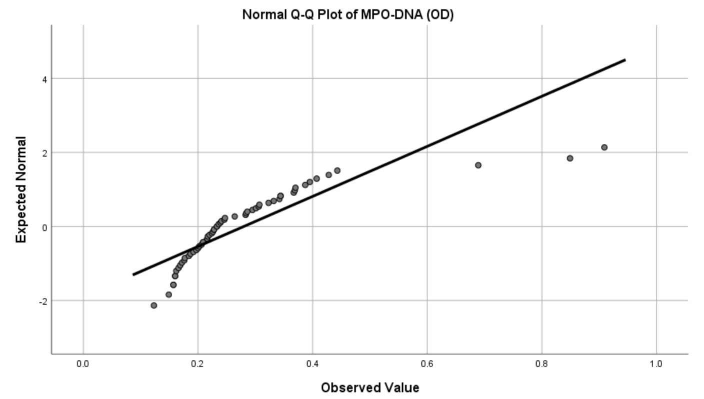	NO
**MPO-DNA day 2**	52	2.33	6.43	0.22	<0.001	0.76	<0.001	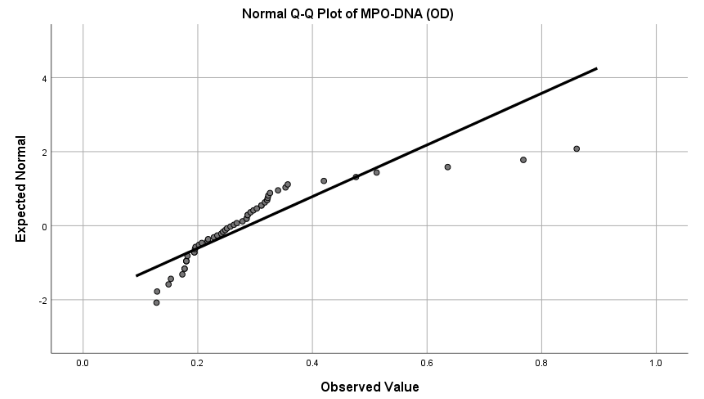	NO

**MPO-DNA day 5**	59	3.01	11.66	0.22	<0.001	0.71	<0.001	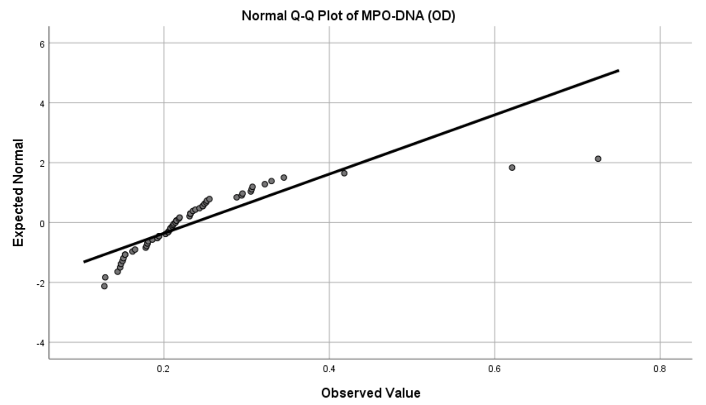	NO
**MPO-DNA day 42**	51	2.76	7.90	0.25	<0.001	0.65	<0.001	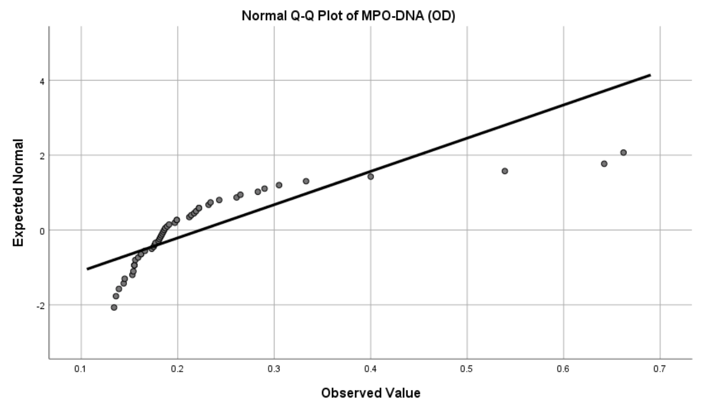	NO
**CitH3 BL**	61	2.06	4.51	0.15	0.002	0.84	<0.001	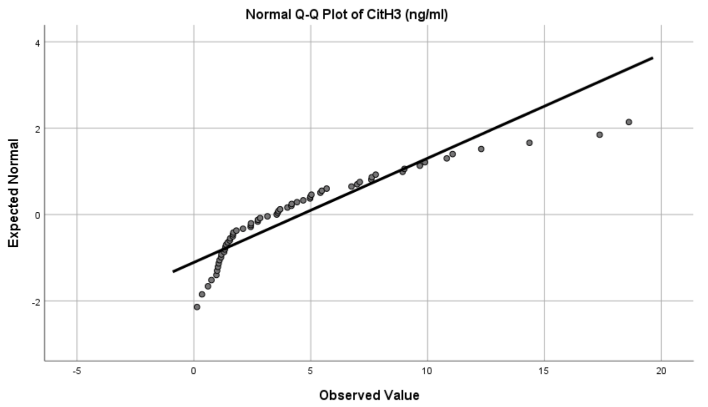	NO

**CitH3 day 1**	60	1.08	0.43	0.18	<0.001	0.76	<0.001	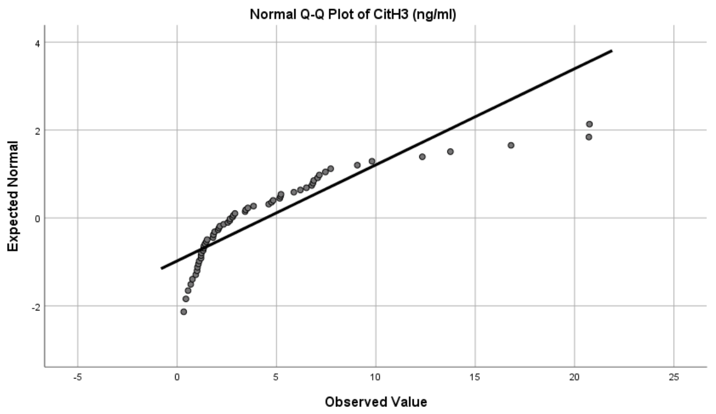	NO
**CitH3 day 2**	52	1.61	2.45	0.17	<0.001	0.88	<0.001	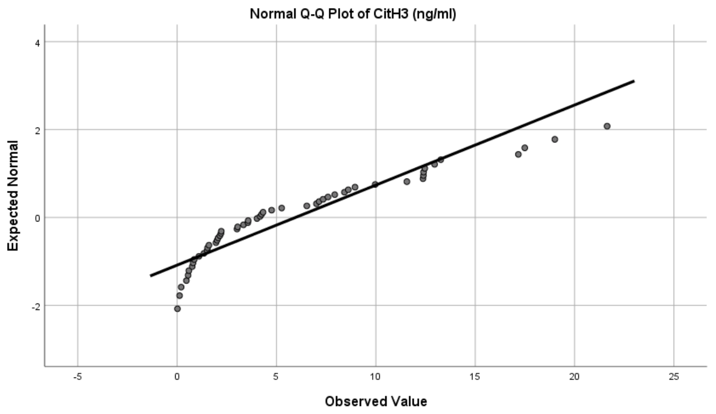	NO
**CitH3 day 5**	59	2.08	4.68	0.18	<0.001	0.81	<0.001	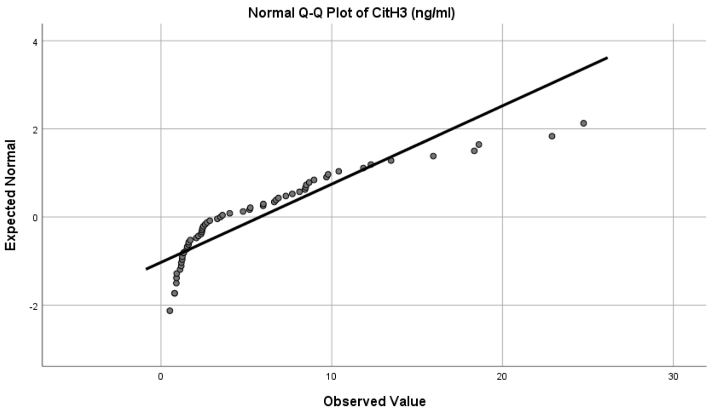	NO

**CitH3 day 42**	51	1.53	2.27	0.21	<0.001	0.77	<0.001	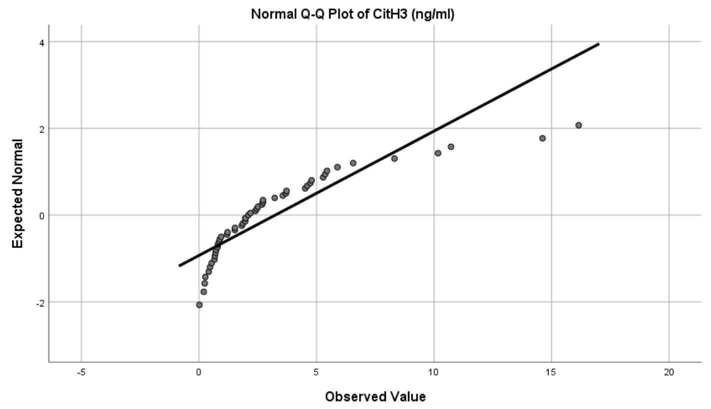	NO
**IL-8 BL**	60	3.99	19.06	0.24	<0.001	0.57	<0.001	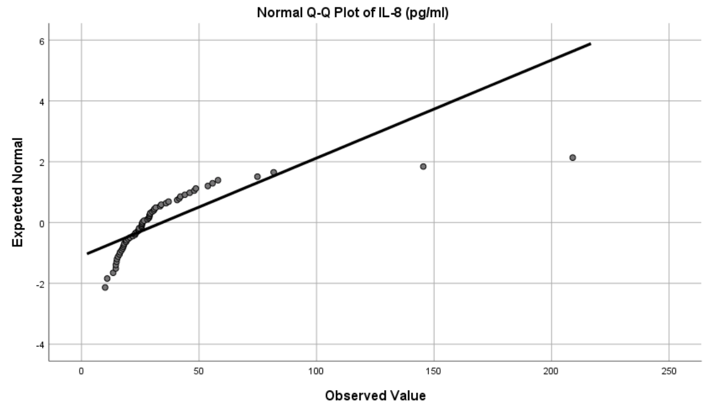	NO
**IL-8 day 1**	59	1.38	1.94	0.17	<0.001	0.88	<0.001	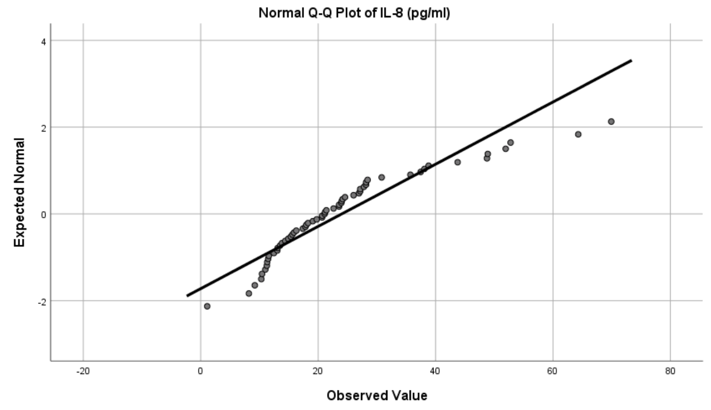	NO

**IL-8 day 2**	52	2.33	7.60	0.15	0.007	0.80	<0.001	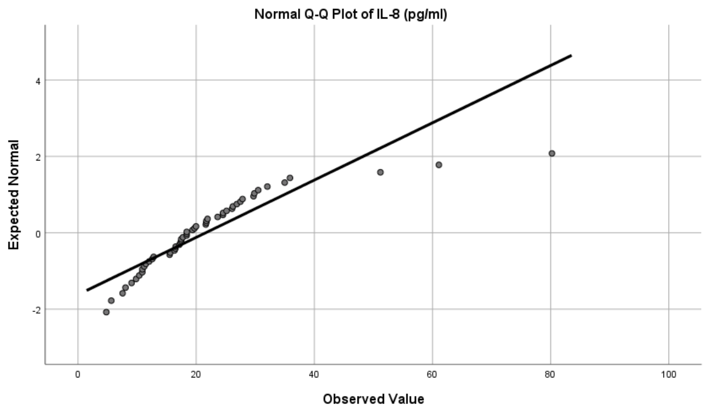	NO
**IL-8 day 5**	59	0.96	1.06	0.11	0.08	0.94	0.007	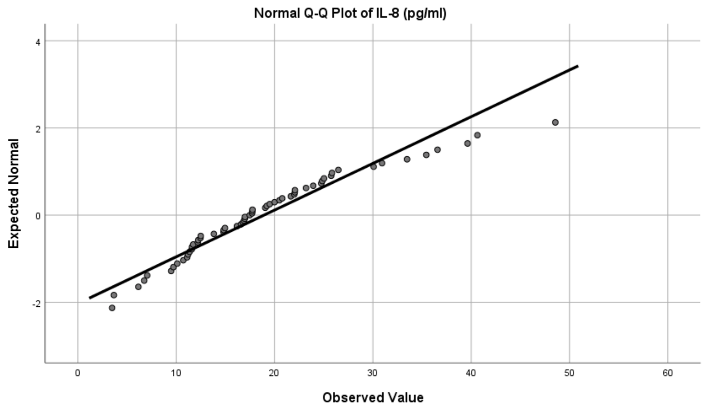	NO
**IL-8 day 42**	53	1.81	4.64	0.18	<0.001	0.84	<0.001	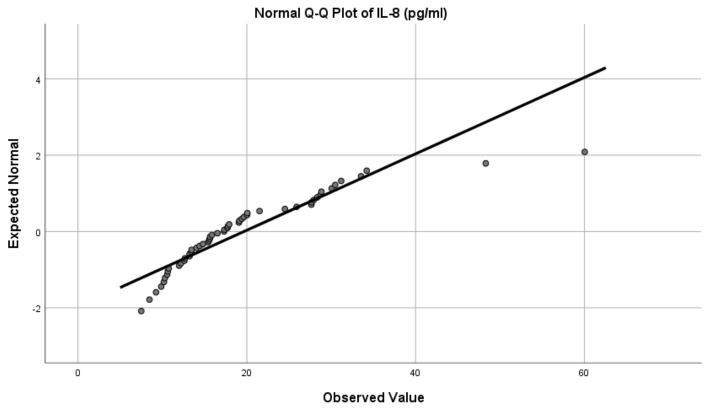	NO

* Kolmogorov-Smirnov test
** Shapiro-Wilk test

2.1 WMSI

2.1.1 Check the model fit

For an example model (for WMSI ~ log.dsDNA without any of the potential confounders), check:

- Do the residuals follow a normal distribution?
- Are the residuals homoscedastic?

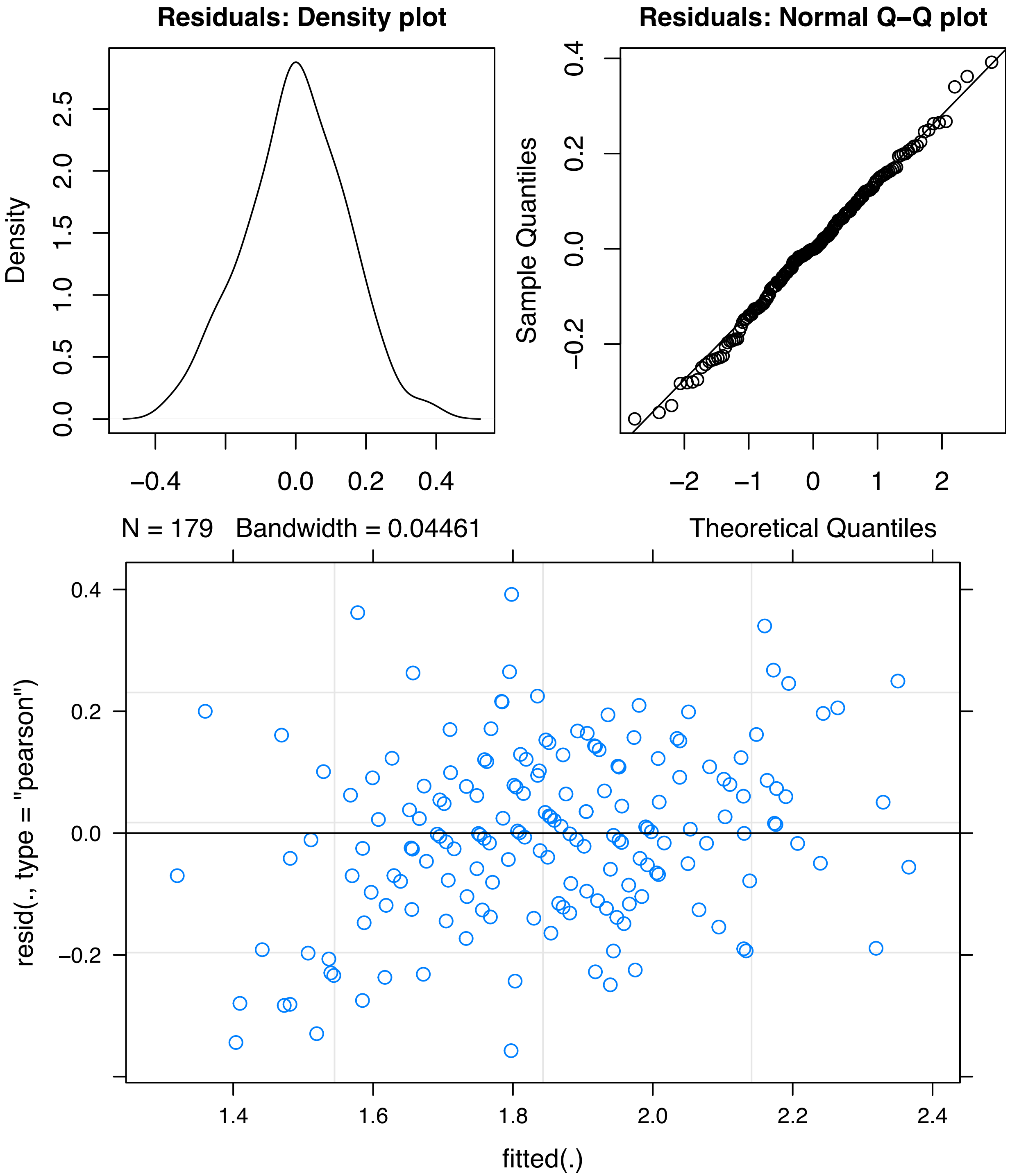

2.1.2 Check for confounders

```
## With NET variable log.dsDNA :
##                 Estimate Std. Error      df t value Pr(>|t|)
## (Intercept)        2.341      0.774 160.334   3.024    0.003
## log.dsDNA         -0.065      0.129 148.651  -0.502    0.616
## day               -0.317      0.210 130.348  -1.512    0.133
## AGE                0.000      0.003  58.622   0.044    0.965
## SEX1              -0.041      0.070  56.809  -0.586    0.560
## KILLIP0           -0.007      0.043  56.199  -0.157    0.876
## log.dsDNA:day      0.047      0.035 130.587   1.329    0.186
##                 Estimate Std. Error      df t value Pr(>|t|)
## (Intercept)        2.401      0.792 150.178   3.032    0.003
## log.dsDNA         -0.065      0.134 140.667  -0.483    0.630
## day               -0.293      0.215 125.099  -1.360    0.176
## AGE               -0.001      0.003  57.266  -0.235    0.815
## SEX1              -0.038      0.072  53.887  -0.530    0.598
## RØYK_V01          -0.069      0.071  56.123  -0.967    0.338
## log.dsDNA:day      0.043      0.036 125.307   1.180    0.240
##                 Estimate Std. Error      df t value Pr(>|t|)
## (Intercept)        2.317      0.768 156.142   3.018    0.003
## log.dsDNA         -0.063      0.129 148.078  -0.483    0.630
## day               -0.316      0.210 130.561  -1.506    0.135
## AGE                0.001      0.003  56.907   0.193    0.848
## SEX1              -0.053      0.079  55.751  -0.671    0.505
## KREAT              0.000      0.001  56.172  -0.335    0.739
## log.dsDNA:day      0.046      0.035 130.789   1.322    0.188
##                 Estimate Std. Error      df t value Pr(>|t|)
## (Intercept)        2.749      0.847 158.821   3.246    0.001
## log.dsDNA         -0.129      0.139 149.268  -0.926    0.356
## day               -0.379      0.217 128.640  -1.748    0.083
## AGE               -0.001      0.003  56.782  -0.208    0.836
## SEX1              -0.036      0.070  56.389  -0.512    0.610
## LEUK_V0           -0.001      0.008  56.225  -0.106    0.916
## log.dsDNA:day      0.057      0.036 128.859   1.573    0.118
##                 Estimate Std. Error      df t value Pr(>|t|)
## (Intercept)        2.308      0.767 157.253   3.011    0.003
## log.dsDNA         -0.073      0.129 148.418  -0.565    0.573
## day               -0.315      0.210 130.820  -1.505    0.135
## AGE                0.000      0.003  58.551   0.081    0.935
## SEX1              -0.031      0.071  56.587  -0.436    0.664
## TnT                0.000      0.000  57.142   0.898    0.373
## log.dsDNA:day      0.046      0.035 131.050   1.322    0.188
##
## With NET variable log.CitH3 :
##                 Estimate Std. Error      df t value Pr(>|t|)
## (Intercept)        1.929      0.208  56.095   9.261    0.000
## log.CitH3         -0.004      0.023 147.936  -0.172    0.864
## day               -0.034      0.010 121.775  -3.257    0.001
## AGE                0.000      0.003  55.201   0.140    0.889
## SEX1              -0.039      0.069  54.829  -0.563    0.576
## KILLIP0           -0.002      0.043  56.108  -0.039    0.969
## log.CitH3:day     -0.003      0.007 125.661  -0.402    0.689
##                 Estimate Std. Error      df t value Pr(>|t|)
```

```
## (Intercept)      2.003      0.190  56.044  10.530    0.000
## log.CitH3       -0.006      0.023 142.867  -0.277    0.782
## day             -0.035      0.010 116.146  -3.453    0.001
## AGE              0.000      0.003  53.956  -0.150    0.881
## SEX1            -0.036      0.071  52.855  -0.509    0.613
## RØYK_V01        -0.061      0.070  54.755  -0.873    0.386
## log.CitH3:day   -0.002      0.007 120.010  -0.287    0.775
##               Estimate Std. Error      df t value Pr(>|t|)
## (Intercept)      1.933      0.169  57.541  11.414    0.000
## log.CitH3       -0.004      0.023 149.326  -0.163    0.871
## day             -0.034      0.010 121.646  -3.247    0.002
## AGE              0.001      0.003  55.206   0.257    0.798
## SEX1            -0.049      0.077  54.701  -0.637    0.527
## KREAT            0.000      0.001  55.161  -0.300    0.766
## log.CitH3:day   -0.003      0.007 125.465  -0.413    0.680
##               Estimate Std. Error      df t value Pr(>|t|)
## (Intercept)      1.968      0.213  54.884   9.233    0.000
## log.CitH3       -0.001      0.023 146.728  -0.034    0.973
## day             -0.032      0.010 119.962  -3.109    0.002
## AGE              0.000      0.003  54.088  -0.145    0.885
## SEX1            -0.028      0.068  54.264  -0.409    0.684
## LEUK_V0         -0.001      0.008  55.489  -0.086    0.932
## log.CitH3:day   -0.004      0.007 124.268  -0.580    0.563
##               Estimate Std. Error      df t value Pr(>|t|)
## (Intercept)      1.865      0.178  58.756  10.453    0.000
## log.CitH3       -0.003      0.023 149.652  -0.132    0.895
## day             -0.034      0.010 121.994  -3.278    0.001
## AGE              0.000      0.002  55.415   0.150    0.881
## SEX1            -0.028      0.069  55.002  -0.404    0.688
## TnT              0.000      0.000  56.568   0.905    0.369
## log.CitH3:day   -0.003      0.007 126.084  -0.386    0.700
##
## With NET variable log.MPODNA :
##                Estimate Std. Error      df t value Pr(>|t|)
## (Intercept)       1.934      0.224  70.160   8.646    0.000
## log.MPODNA        0.002      0.056 156.407   0.042    0.967
## day              -0.027      0.028 125.466  -0.976    0.331
## AGE               0.000      0.003  56.253   0.147    0.884
## SEX1             -0.040      0.069  56.055  -0.577    0.567
## KILLIP0          -0.004      0.043  56.579  -0.101    0.920
## log.MPODNA:day    0.007      0.018 124.305   0.380    0.705
##                Estimate Std. Error      df t value Pr(>|t|)
## (Intercept)       2.000      0.204  69.175   9.821    0.000
## log.MPODNA       -0.002      0.055 149.297  -0.040    0.968
## day              -0.021      0.027 118.182  -0.786    0.433
## AGE               0.000      0.003  54.515  -0.164    0.870
## SEX1             -0.038      0.071  53.526  -0.530    0.598
## RØYK_V01         -0.071      0.071  56.532  -1.004    0.320
## log.MPODNA:day    0.011      0.018 117.212   0.610    0.543
##                Estimate Std. Error      df t value Pr(>|t|)
## (Intercept)       1.928      0.183  73.075  10.552    0.000
## log.MPODNA        0.001      0.056 156.784   0.024    0.981
## day              -0.027      0.028 125.439  -0.973    0.332
## AGE               0.001      0.003  56.299   0.267    0.791
```

```
## SEX1             -0.050      0.078  56.258  -0.644    0.522
## KREAT             0.000      0.001  56.084  -0.293    0.771
## log.MPODNA:day    0.007      0.018 124.242   0.385    0.701
##                Estimate Std. Error      df t value Pr(>|t|)
## (Intercept)       1.967      0.229  68.131   8.590    0.000
## log.MPODNA       -0.001      0.056 155.885  -0.014    0.989
## day              -0.031      0.030 125.869  -1.050    0.296
## AGE               0.000      0.003  54.794  -0.148    0.883
## SEX1             -0.030      0.069  55.123  -0.443    0.660
## LEUK_V0          -0.001      0.008  56.635  -0.076    0.940
## log.MPODNA:day    0.004      0.020 123.576   0.206    0.837
##                Estimate Std. Error      df t value Pr(>|t|)
## (Intercept)       1.857      0.192  74.121   9.656    0.000
## log.MPODNA       -0.001      0.056 156.876  -0.020    0.984
## day              -0.027      0.028 125.655  -0.996    0.321
## AGE               0.000      0.003  56.404   0.163    0.871
## SEX1             -0.029      0.069  56.058  -0.416    0.679
## TnT               0.000      0.000  57.370   0.929    0.357
## log.MPODNA:day    0.007      0.018 124.516   0.366    0.715
```

2.1.3 Summarise all final models (no confounders)

log.dsDNA:

```
## Linear mixed model fit by REML. t-tests use Satterthwaite's method [
## lmerModLmerTest]
## Formula: as.formula(fma)
##    Data: dat
##
## REML criterion at convergence: -0.8
##
## Scaled residuals:
##     Min      1Q  Median      3Q     Max
## -2.0997 -0.5317 -0.0040  0.5696  2.3028
##
## Random effects:
##  Groups   Name        Variance Std.Dev.
##  ID       (Intercept) 0.04832  0.2198
##  Residual             0.02897  0.1702
## Number of obs: 179, groups:  ID, 61
##
## Fixed effects:
##               Estimate Std. Error        df t value Pr(>|t|)
## (Intercept)     2.24355    0.75315 156.01538   2.979  0.00336 **
## log.dsDNA      -0.05225    0.12666 155.74092  -0.413  0.68049
## day            -0.30404    0.20911 131.77257  -1.454  0.14833
## log.dsDNA:day   0.04441    0.03498 132.02646   1.269  0.20650
## ---
## Signif. codes:  0 '***' 0.001 '**' 0.01 '*' 0.05 '.' 0.1 ' ' 1
##
## Correlation of Fixed Effects:
##             (Intr) lg.DNA day
## log.dsDNA   -0.999
## day         -0.680  0.680
## lg.dsDNA:dy  0.686 -0.686 -1.000
```

log.CitH3:

```
## Linear mixed model fit by REML. t-tests use Satterthwaite's method [
## lmerModLmerTest]
## Formula: as.formula(fma)
##    Data: dat
##
## REML criterion at convergence: 7.2
##
## Scaled residuals:
##      Min       1Q   Median       3Q      Max
## -2.22841 -0.52055 -0.01067  0.60425  2.45356
##
## Random effects:
##  Groups   Name        Variance Std.Dev.
##  ID       (Intercept) 0.04624  0.2150
##  Residual             0.02981  0.1727
## Number of obs: 179, groups:  ID, 61
##
## Fixed effects:
##                Estimate Std. Error         df t value Pr(>|t|)
## (Intercept)     1.936718   0.041069 131.887588  47.158  < 2e-16 ***
## log.CitH3      -0.004523   0.023257 151.504920  -0.194  0.84605
## day            -0.033525   0.010340 122.391335  -3.242  0.00153 **
## log.CitH3:day  -0.002917   0.006850 126.938271  -0.426  0.67091
## ---
## Signif. codes:  0 '***' 0.001 '**' 0.01 '*' 0.05 '.' 0.1 ' ' 1
##
## Correlation of Fixed Effects:
##             (Intr) lg.CH3 day
## log.CitH3   -0.606
## day         -0.436  0.423
## log.CtH3:dy  0.384 -0.611 -0.808
```

log.MPODNA:

```
## Linear mixed model fit by REML. t-tests use Satterthwaite's method [
## lmerModLmerTest]
## Formula: as.formula(fma)
##    Data: dat
##
## REML criterion at convergence: 3.5
##
## Scaled residuals:
##      Min       1Q   Median       3Q      Max
## -2.22581 -0.48276  0.00195  0.61079  2.39270
##
## Random effects:
##  Groups   Name        Variance Std.Dev.
##  ID       (Intercept) 0.04700  0.2168
##  Residual             0.02969  0.1723
## Number of obs: 179, groups:  ID, 61
##
## Fixed effects:
##                  Estimate Std. Error         df t value Pr(>|t|)
## (Intercept)      1.938005   0.079411 174.203038  24.405   <2e-16 ***
## log.MPODNA       0.004663   0.055492 159.776526   0.084    0.933
## day             -0.027170   0.027508 126.037352  -0.988    0.325
## log.MPODNA:day   0.006769   0.018379 124.858819   0.368    0.713
## ---
## Signif. codes:  0 '***' 0.001 '**' 0.01 '*' 0.05 '.' 0.1 ' ' 1
##
## Correlation of Fixed Effects:
##             (Intr) lg.MPODNA day
## log.MPODNA   0.910
## day         -0.362 -0.349
## lg.MPODNA:d -0.405 -0.432     0.973
```

2.2 GLS

2.2.1 Check the model fit

For an example model (for GLS ~ log.dsDNA without any of the potential confounders), check:

- Do the residuals follow a normal distribution?
- Are the residuals homoscedastic?

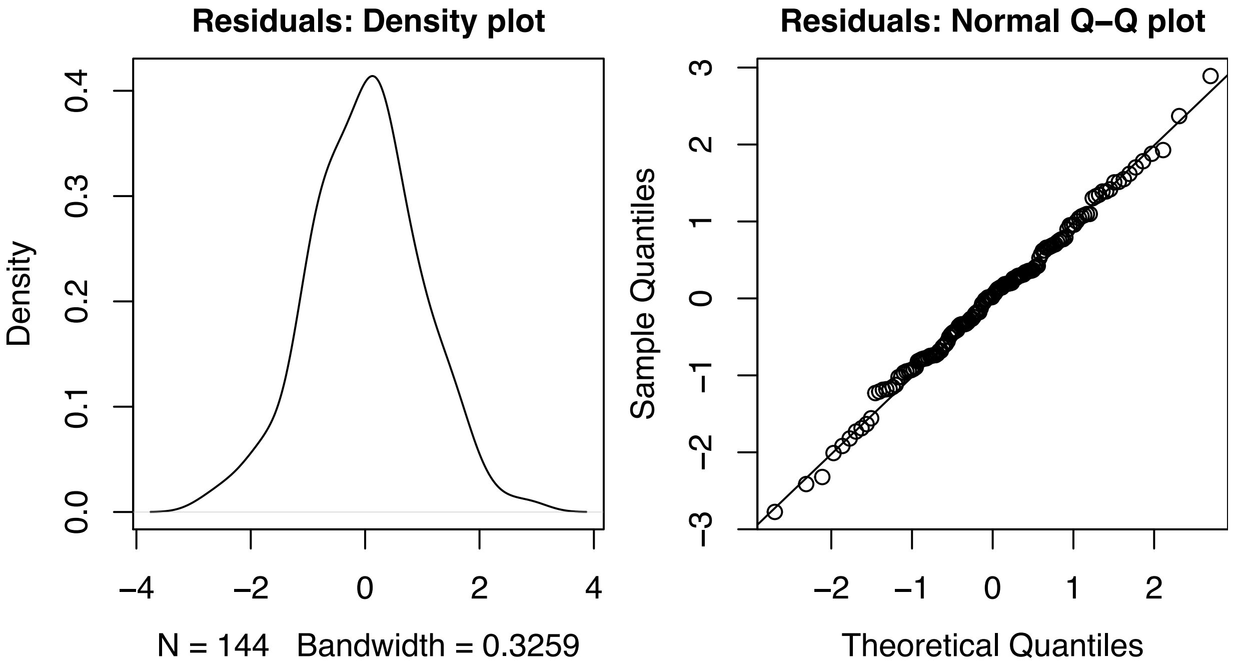

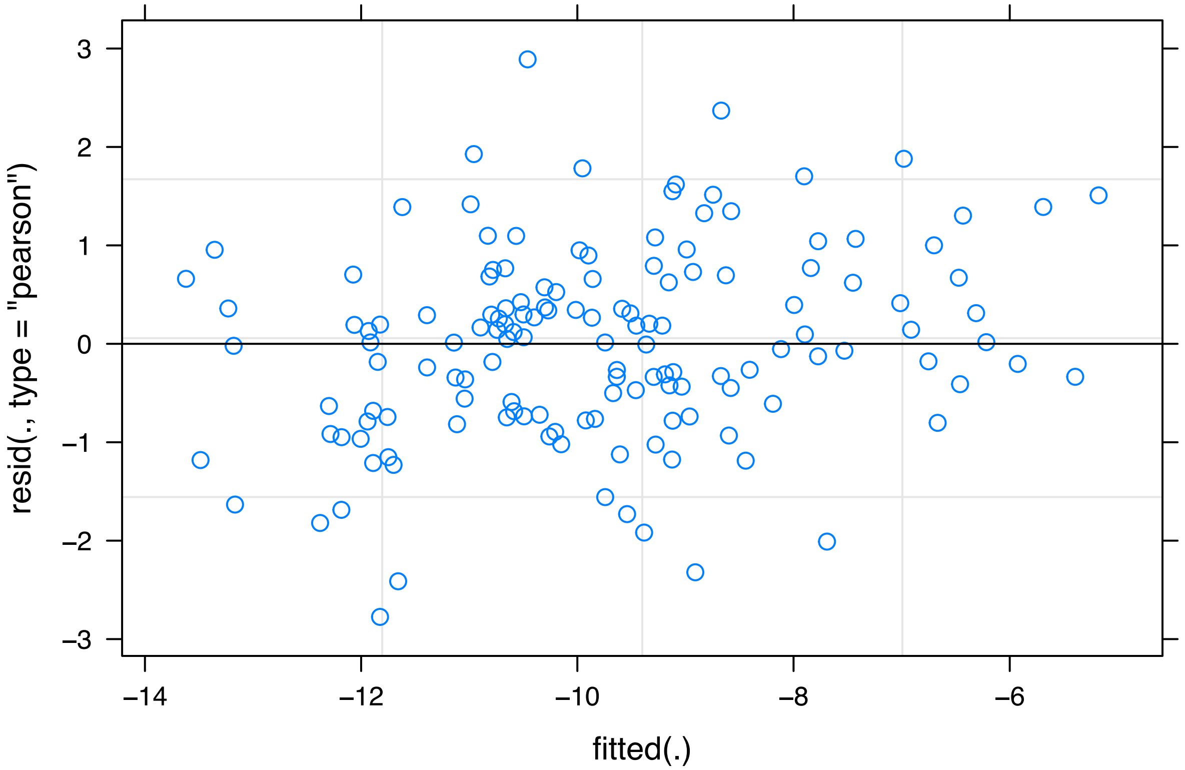

2.2.2 Check for confounders

```
## With NET variable log.dsDNA :
##                Estimate Std. Error      df t value Pr(>|t|)
## (Intercept)     -11.653      6.368 126.074  -1.830    0.070
## log.dsDNA         0.780      1.042 113.031   0.749    0.456
## day               0.477      1.757 104.014   0.271    0.787
## AGE              -0.026      0.025  44.949  -1.062    0.294
## SEX1             -1.226      0.623  44.677  -1.967    0.055
## KILLIP0          -0.026      0.382  44.876  -0.068    0.946
## log.dsDNA:day    -0.132      0.295 104.087  -0.449    0.654
##                Estimate Std. Error      df t value Pr(>|t|)
## (Intercept)     -11.704      6.308 122.351  -1.855    0.066
## log.dsDNA         0.800      1.048 111.540   0.763    0.447
## day               0.472      1.758 103.888   0.269    0.789
## AGE              -0.028      0.026  46.208  -1.069    0.291
## SEX1             -1.224      0.622  44.614  -1.968    0.055
## RØYK_V01         -0.113      0.588  46.790  -0.192    0.849
## log.dsDNA:day    -0.132      0.295 103.971  -0.447    0.656
##                Estimate Std. Error      df t value Pr(>|t|)
## (Intercept)     -12.126      6.274 123.390  -1.933    0.056
## log.dsDNA         0.678      1.039 114.106   0.652    0.515
## day               0.403      1.754 104.743   0.230    0.819
## AGE              -0.036      0.024  44.659  -1.492    0.143
## SEX1             -0.758      0.657  44.138  -1.154    0.255
## KREAT             0.018      0.010  44.379   1.767    0.084
## log.dsDNA:day    -0.120      0.294 104.811  -0.407    0.685
##                Estimate Std. Error      df t value Pr(>|t|)
## (Intercept)     -14.988      7.215 125.243  -2.077    0.040
## log.dsDNA         1.151      1.143 115.455   1.007    0.316
## day               0.841      1.828 103.506   0.460    0.646
## AGE              -0.025      0.025  44.139  -1.021    0.313
## SEX1             -1.032      0.586  44.174  -1.761    0.085
## LEUK_V0           0.062      0.075  45.326   0.824    0.415
## log.dsDNA:day    -0.193      0.307 103.570  -0.628    0.531
##                Estimate Std. Error      df t value Pr(>|t|)
## (Intercept)     -12.541      6.253 124.345  -2.005    0.047
## log.dsDNA         0.671      1.034 115.101   0.648    0.518
## day               0.470      1.747 105.781   0.269    0.788
## AGE              -0.021      0.023  45.874  -0.888    0.379
## SEX1             -1.038      0.600  45.056  -1.729    0.091
## TnT               0.000      0.000  45.542   2.204    0.033
## log.dsDNA:day    -0.131      0.293 105.858  -0.447    0.656
##
## With NET variable log.CitH3 :
##                Estimate Std. Error      df t value Pr(>|t|)
## (Intercept)      -7.058      1.899  43.841  -3.716    0.001
## log.CitH3        -0.045      0.189 111.927  -0.236    0.814
## day              -0.210      0.080  93.294  -2.635    0.010
## AGE              -0.027      0.024  43.884  -1.107    0.274
## SEX1             -1.174      0.613  44.157  -1.914    0.062
## KILLIP0           0.015      0.377  44.677   0.039    0.969
## log.CitH3:day    -0.068      0.052  96.362  -1.312    0.193
##                Estimate Std. Error      df t value Pr(>|t|)
```

```
## (Intercept)     -7.071      1.702  46.019  -4.156    0.000
## log.CitH3       -0.044      0.189 113.084  -0.234    0.816
## day             -0.209      0.080  92.541  -2.623    0.010
## AGE             -0.026      0.025  44.369  -1.046    0.301
## SEX1            -1.175      0.612  44.104  -1.919    0.062
## RØYK_V01         0.045      0.574  44.861   0.078    0.938
## log.CitH3:day   -0.069      0.052  95.659  -1.316    0.191
##               Estimate Std. Error      df t value Pr(>|t|)
## (Intercept)     -7.981      1.583  45.114  -5.043    0.000
## log.CitH3       -0.076      0.188 113.588  -0.405    0.686
## day             -0.216      0.080  93.238  -2.706    0.008
## AGE             -0.038      0.024  43.613  -1.583    0.121
## SEX1            -0.694      0.645  43.574  -1.076    0.288
## KREAT            0.018      0.010  43.700   1.831    0.074
## log.CitH3:day   -0.062      0.052  96.178  -1.193    0.236
##               Estimate Std. Error      df t value Pr(>|t|)
## (Intercept)     -7.934      1.956  43.531  -4.056    0.000
## log.CitH3       -0.036      0.188 113.119  -0.192    0.848
## day             -0.194      0.080  92.824  -2.416    0.018
## AGE             -0.027      0.024  43.191  -1.118    0.270
## SEX1            -0.979      0.571  43.689  -1.713    0.094
## LEUK_V0          0.054      0.072  43.813   0.738    0.464
## log.CitH3:day   -0.076      0.053  96.505  -1.452    0.150
##               Estimate Std. Error      df t value Pr(>|t|)
## (Intercept)     -8.423      1.619  47.234  -5.202    0.000
## log.CitH3       -0.040      0.187 115.273  -0.212    0.832
## day             -0.215      0.079  94.439  -2.704    0.008
## AGE             -0.022      0.023  45.051  -0.957    0.343
## SEX1            -0.994      0.592  44.621  -1.679    0.100
## TnT              0.000      0.000  45.048   2.165    0.036
## log.CitH3:day   -0.065      0.052  97.822  -1.264    0.209
##
## With NET variable log.MPODNA :
##                 Estimate Std. Error      df t value Pr(>|t|)
## (Intercept)       -7.301      2.032  51.287  -3.593    0.001
## log.MPODNA        -0.120      0.420 118.940  -0.285    0.777
## day               -0.254      0.198  94.479  -1.288    0.201
## AGE               -0.025      0.025  43.966  -0.996    0.325
## SEX1              -1.272      0.626  43.916  -2.033    0.048
## KILLIP0           -0.009      0.384  44.437  -0.024    0.981
## log.MPODNA:day     0.038      0.135  93.780   0.280    0.780
##                 Estimate Std. Error      df t value Pr(>|t|)
## (Intercept)       -7.251      1.822  53.767  -3.980    0.000
## log.MPODNA        -0.118      0.420 118.984  -0.280    0.780
## day               -0.253      0.198  93.071  -1.274    0.206
## AGE               -0.025      0.026  44.435  -0.986    0.330
## SEX1              -1.271      0.624  43.800  -2.036    0.048
## RØYK_V01          -0.059      0.588  45.457  -0.101    0.920
## log.MPODNA:day     0.039      0.135  92.413   0.288    0.774
##                 Estimate Std. Error      df t value Pr(>|t|)
## (Intercept)       -8.279      1.693  52.380  -4.891    0.000
## log.MPODNA        -0.076      0.418 120.509  -0.181    0.857
## day               -0.260      0.197  94.703  -1.317    0.191
## AGE               -0.035      0.024  43.741  -1.449    0.154
```

```
## SEX1             -0.788      0.659  43.597  -1.195    0.239
## KREAT             0.018      0.010  43.570   1.794    0.080
## log.MPODNA:day    0.032      0.135  93.897   0.239    0.812
##                Estimate Std. Error      df t value Pr(>|t|)
## (Intercept)      -8.195      2.150  53.537  -3.812    0.000
## log.MPODNA       -0.102      0.423 120.387  -0.241    0.810
## day              -0.234      0.215  96.672  -1.086    0.280
## AGE              -0.025      0.025  43.862  -1.003    0.321
## SEX1             -1.084      0.589  43.987  -1.841    0.072
## LEUK_V0           0.054      0.075  45.079   0.720    0.475
## log.MPODNA:day    0.046      0.144  94.942   0.319    0.750
##                Estimate Std. Error      df t value Pr(>|t|)
## (Intercept)      -8.867      1.733  55.681  -5.118    0.000
## log.MPODNA       -0.165      0.416 121.148  -0.397    0.692
## day              -0.259      0.197  95.901  -1.319    0.190
## AGE              -0.020      0.024  45.103  -0.832    0.410
## SEX1             -1.077      0.602  44.558  -1.788    0.081
## TnT               0.000      0.000  45.374   2.250    0.029
## log.MPODNA:day    0.036      0.134  95.191   0.269    0.788
```

2.2.3 Summarise all final models (no confounders)

log.dsDNA:

```
## Linear mixed model fit by REML. t-tests use Satterthwaite's method [
## lmerModLmerTest]
## Formula: as.formula(fma)
##    Data: dat
##
## REML criterion at convergence: 563.9
##
## Scaled residuals:
##      Min       1Q   Median       3Q      Max
## -2.33264 -0.58340  0.02769  0.55400  2.42963
##
## Random effects:
##  Groups   Name        Variance Std.Dev.
##  ID       (Intercept) 3.481    1.866
##  Residual             1.415    1.189
## Number of obs: 144, groups:  ID, 51
##
## Fixed effects:
##               Estimate Std. Error       df t value Pr(>|t|)
## (Intercept)   -14.1548     6.1920 113.6712  -2.286   0.0241 *
## log.dsDNA       0.8544     1.0414 113.3206   0.820   0.4137
## day             0.6208     1.7591 103.6545   0.353   0.7249
## log.dsDNA:day  -0.1566     0.2949 103.7294  -0.531   0.5967
## ---
## Signif. codes:  0 '***' 0.001 '**' 0.01 '*' 0.05 '.' 0.1 ' ' 1
##
## Correlation of Fixed Effects:
##             (Intr) lg.DNA day
## log.dsDNA   -0.999
## day         -0.678  0.678
## lg.dsDNA:dy  0.682 -0.683 -1.000
```

log.CitH3:

```
## Linear mixed model fit by REML. t-tests use Satterthwaite's method [
## lmerModLmerTest]
## Formula: as.formula(fma)
##    Data: dat
##
## REML criterion at convergence: 567.5
##
## Scaled residuals:
##      Min       1Q   Median       3Q      Max
## -2.40081 -0.57233  0.07182  0.50516  2.36317
##
## Random effects:
##  Groups   Name        Variance Std.Dev.
##  ID       (Intercept) 3.360    1.833
##  Residual             1.392    1.180
## Number of obs: 144, groups:  ID, 51
##
## Fixed effects:
##               Estimate Std. Error        df t value Pr(>|t|)
## (Intercept)   -9.04320    0.35933 102.26670 -25.167   <2e-16 ***
## log.CitH3     -0.04431    0.18910 112.30498  -0.234    0.815
## day           -0.20659    0.07967  93.39517  -2.593    0.011 *
## log.CitH3:day -0.07076    0.05199  96.49800  -1.361    0.177
## ---
## Signif. codes:  0 '***' 0.001 '**' 0.01 '*' 0.05 '.' 0.1 ' ' 1
##
## Correlation of Fixed Effects:
##             (Intr) lg.CH3 day
## log.CitH3   -0.592
## day         -0.413  0.438
## log.CtH3:dy  0.386 -0.630 -0.815
```

log.MPODNA:

```
## Linear mixed model fit by REML. t-tests use Satterthwaite's method [
## lmerModLmerTest]
## Formula: as.formula(fma)
##    Data: dat
##
## REML criterion at convergence: 567.4
##
## Scaled residuals:
##      Min       1Q   Median       3Q      Max
## -2.32394 -0.55145  0.02868  0.52692  2.41617
##
## Random effects:
##  Groups   Name        Variance Std.Dev.
##  ID       (Intercept) 3.530    1.879
##  Residual             1.415    1.189
## Number of obs: 144, groups:  ID, 51
##
## Fixed effects:
##                 Estimate Std. Error        df t value Pr(>|t|)
## (Intercept)     -9.20708    0.61056 139.70924 -15.080   <2e-16 ***
## log.MPODNA      -0.09901    0.42116 118.73502  -0.235    0.815
## day             -0.25906    0.19748  94.61704  -1.312    0.193
## log.MPODNA:day   0.03421    0.13453  93.91229   0.254    0.800
## ---
## Signif. codes:  0 '***' 0.001 '**' 0.01 '*' 0.05 '.' 0.1 ' ' 1
##
## Correlation of Fixed Effects:
##             (Intr) lg.MPODNA day
## log.MPODNA   0.875
## day         -0.331 -0.328
## lg.MPODNA:d -0.372 -0.414     0.969
```

2.3 EF

2.3.1 Check the model fit

For an example model (for EF ~ log.dsDNA without any of the potential confounders), check:

- Do the residuals follow a normal distribution?
- Are the residuals homoscedastic?

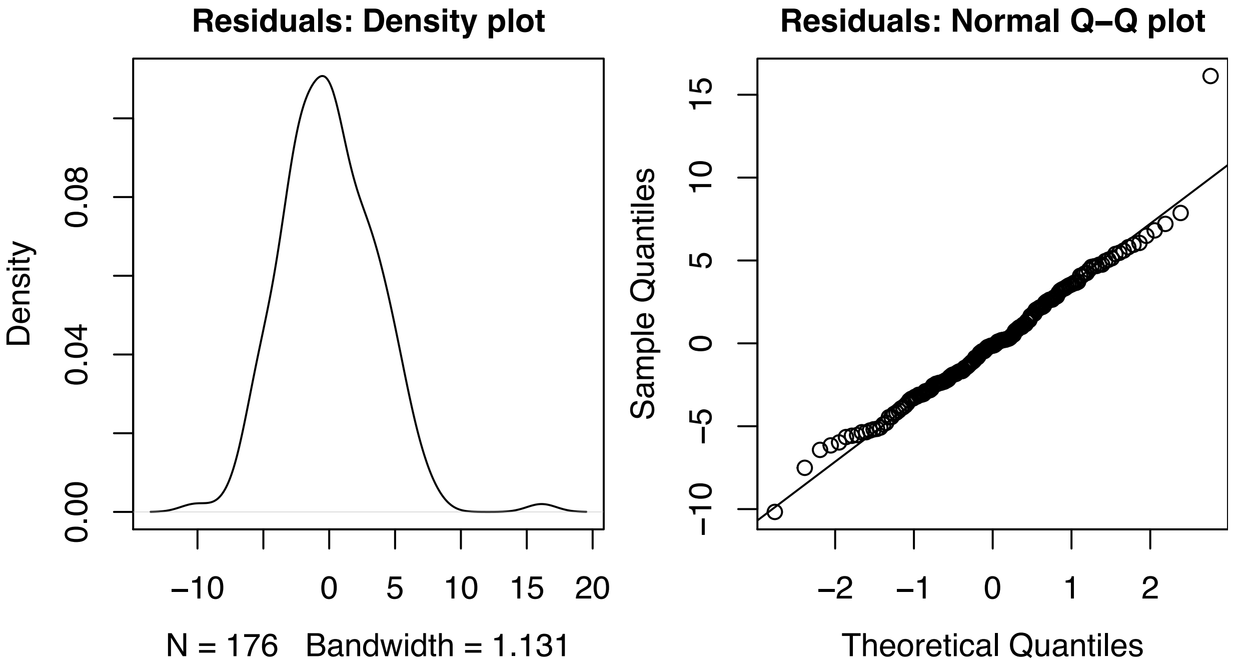

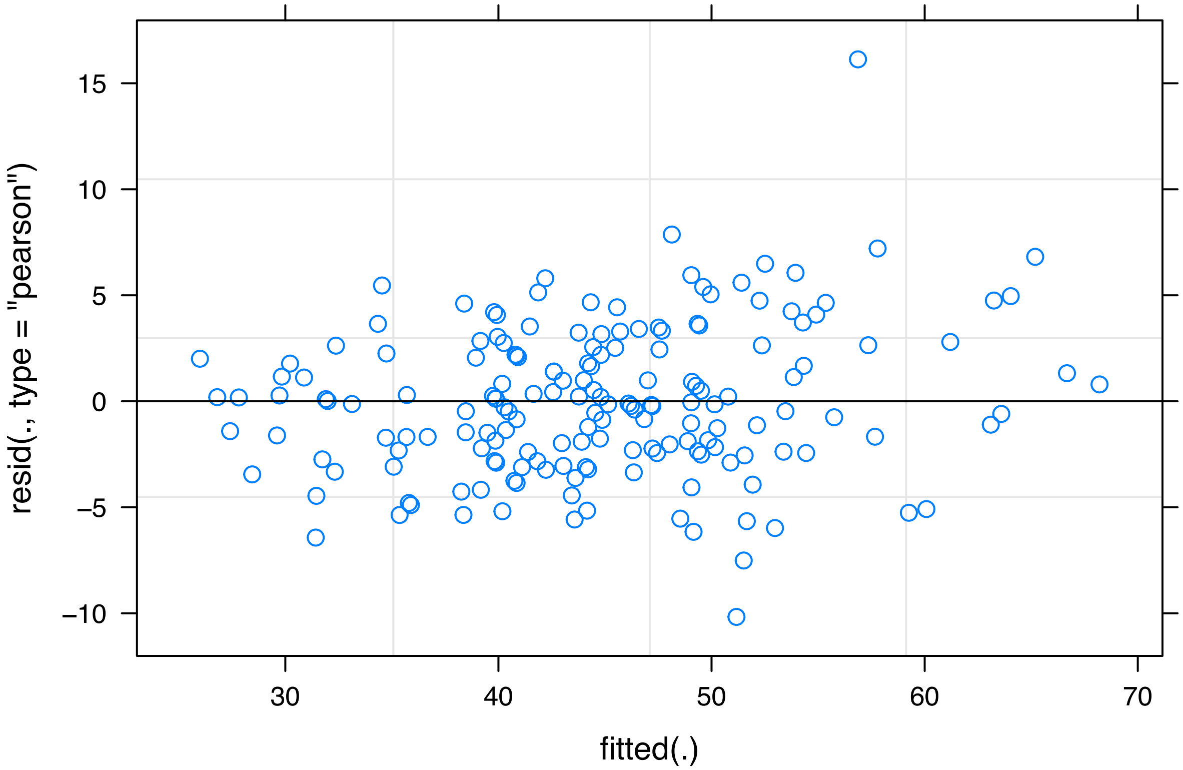

2.3.2 Check for confounders

```
## With NET variable log.dsDNA :
##               Estimate Std. Error      df t value Pr(>|t|)
## (Intercept)     65.871     21.160 157.647   3.113    0.002
## log.dsDNA       -3.613      3.413 131.490  -1.058    0.292
## day              4.655      5.495 122.656   0.847    0.399
## AGE             -0.005      0.096  56.903  -0.050    0.960
## SEX1             2.727      2.535  56.091   1.076    0.287
## KILLIP0         -0.912      1.577  55.717  -0.578    0.565
## log.dsDNA:day   -0.609      0.919 122.731  -0.663    0.509
##               Estimate Std. Error      df t value Pr(>|t|)
## (Intercept)     55.629     21.973 146.027   2.532    0.012
## log.dsDNA       -3.204      3.621 125.749  -0.885    0.378
## day              5.905      5.823 118.445   1.014    0.313
## AGE              0.063      0.104  55.411   0.608    0.546
## SEX1             2.296      2.603  53.412   0.882    0.382
## RØYK_V01         3.036      2.597  54.948   1.169    0.248
## log.dsDNA:day   -0.819      0.974 118.513  -0.841    0.402
##               Estimate Std. Error      df t value Pr(>|t|)
## (Intercept)     63.347     20.729 149.569   3.056    0.003
## log.dsDNA       -3.632      3.418 130.917  -1.063    0.290
## day              4.696      5.496 122.633   0.854    0.395
## AGE              0.009      0.104  55.997   0.086    0.931
## SEX1             2.596      2.862  55.488   0.907    0.368
## KREAT           -0.005      0.038  55.755  -0.143    0.887
## log.dsDNA:day   -0.616      0.919 122.705  -0.670    0.504
##               Estimate Std. Error      df t value Pr(>|t|)
## (Intercept)     63.509     23.135 152.480   2.745    0.007
## log.dsDNA       -2.961      3.699 131.688  -0.801    0.425
## day              5.800      5.684 120.579   1.020    0.310
## AGE              0.016      0.097  54.967   0.162    0.872
## SEX1             2.385      2.515  54.749   0.948    0.347
## LEUK_V0         -0.364      0.306  54.328  -1.189    0.239
## log.dsDNA:day   -0.803      0.951 120.655  -0.844    0.400
##               Estimate Std. Error      df t value Pr(>|t|)
## (Intercept)     65.056     20.737 151.524   3.137    0.002
## log.dsDNA       -3.499      3.413 131.495  -1.025    0.307
## day              4.692      5.491 122.978   0.855    0.394
## AGE              0.001      0.094  57.053   0.015    0.988
## SEX1             2.315      2.552  56.063   0.907    0.368
## TnT              0.000      0.000  56.347  -1.102    0.275
## log.dsDNA:day   -0.616      0.918 123.050  -0.671    0.504
##
## With NET variable log.CitH3 :
##               Estimate Std. Error      df t value Pr(>|t|)
## (Intercept)     46.517      7.845  55.544   5.929    0.000
## log.CitH3        0.059      0.607 129.872   0.097    0.923
## day              0.483      0.261 116.547   1.851    0.067
## AGE             -0.029      0.095  55.273  -0.302    0.764
## SEX1             2.942      2.520  55.226   1.168    0.248
## KILLIP0         -1.155      1.578  56.100  -0.732    0.467
## log.CitH3:day    0.316      0.173 118.255   1.827    0.070
##               Estimate Std. Error      df t value Pr(>|t|)
```

```
## (Intercept)     38.987      7.202  54.715   5.414    0.000
## log.CitH3        0.078      0.613 126.242   0.128    0.899
## day              0.506      0.266 111.934   1.903    0.060
## AGE              0.031      0.103  53.691   0.302    0.763
## SEX1             2.432      2.606  53.193   0.933    0.355
## RØYK_V01         2.040      2.594  54.245   0.786    0.435
## log.CitH3:day    0.305      0.175 113.790   1.740    0.085
##               Estimate Std. Error      df t value Pr(>|t|)
## (Intercept)     43.303      6.348  56.536   6.821    0.000
## log.CitH3        0.031      0.606 130.592   0.051    0.960
## day              0.483      0.261 116.527   1.847    0.067
## AGE             -0.008      0.103  55.422  -0.079    0.937
## SEX1             2.677      2.858  55.269   0.937    0.353
## KREAT           -0.010      0.038  55.482  -0.259    0.797
## log.CitH3:day    0.318      0.173 118.200   1.837    0.069
##               Estimate Std. Error      df t value Pr(>|t|)
## (Intercept)     47.001      7.765  54.283   6.053    0.000
## log.CitH3       -0.001      0.608 128.628  -0.002    0.998
## day              0.465      0.262 114.714   1.771    0.079
## AGE             -0.007      0.096  53.794  -0.075    0.941
## SEX1             2.533      2.501  53.968   1.013    0.316
## LEUK_V0         -0.330      0.306  54.278  -1.080    0.285
## log.CitH3:day    0.338      0.175 116.668   1.927    0.056
##               Estimate Std. Error      df t value Pr(>|t|)
## (Intercept)     45.699      6.652  57.347   6.870    0.000
## log.CitH3       -0.001      0.605 130.912  -0.002    0.998
## day              0.486      0.261 116.825   1.861    0.065
## AGE             -0.020      0.093  55.594  -0.215    0.831
## SEX1             2.533      2.544  55.458   0.996    0.324
## TnT              0.000      0.000  56.497  -1.112    0.271
## log.CitH3:day    0.317      0.173 118.661   1.835    0.069
##
## With NET variable log.MPODNA :
##                Estimate Std. Error      df t value Pr(>|t|)
## (Intercept)      46.250      8.197  62.292   5.643    0.000
## log.MPODNA       -0.002      1.495 136.415  -0.001    0.999
## day               1.254      0.708 118.442   1.770    0.079
## AGE              -0.028      0.096  55.280  -0.296    0.768
## SEX1              3.136      2.551  55.360   1.229    0.224
## KILLIP0          -1.076      1.593  55.746  -0.675    0.502
## log.MPODNA:day    0.229      0.473 117.648   0.484    0.630
##                Estimate Std. Error      df t value Pr(>|t|)
## (Intercept)      38.773      7.522  61.346   5.155    0.000
## log.MPODNA        0.059      1.514 131.238   0.039    0.969
## day               1.134      0.724 112.887   1.567    0.120
## AGE               0.034      0.104  53.430   0.323    0.748
## SEX1              2.634      2.632  53.004   1.001    0.322
## RØYK_V01          2.326      2.629  54.719   0.885    0.380
## log.MPODNA:day    0.141      0.485 112.227   0.292    0.771
##                Estimate Std. Error      df t value Pr(>|t|)
## (Intercept)      43.219      6.642  63.935   6.506    0.000
## log.MPODNA       -0.053      1.494 136.474  -0.036    0.972
## day               1.254      0.708 118.347   1.770    0.079
## AGE              -0.007      0.104  55.318  -0.070    0.945
```

```
## SEX1              2.819      2.892  55.457   0.975    0.334
## KREAT            -0.011      0.038  55.292  -0.287    0.775
## log.MPODNA:day    0.230      0.473 117.538   0.487    0.627
##                Estimate Std. Error      df t value Pr(>|t|)
## (Intercept)      47.150      8.159  61.447   5.779    0.000
## log.MPODNA        0.180      1.512 136.346   0.119    0.906
## day               1.360      0.772 118.409   1.761    0.081
## AGE              -0.007      0.097  53.622  -0.072    0.943
## SEX1              2.761      2.535  53.926   1.089    0.281
## LEUK_V0          -0.335      0.310  54.456  -1.080    0.285
## log.MPODNA:day    0.290      0.508 116.907   0.571    0.569
##                Estimate Std. Error      df t value Pr(>|t|)
## (Intercept)      45.927      6.947  64.811   6.611    0.000
## log.MPODNA        0.027      1.493 136.857   0.018    0.986
## day               1.264      0.708 118.674   1.785    0.077
## AGE              -0.020      0.094  55.525  -0.217    0.829
## SEX1              2.670      2.566  55.445   1.041    0.302
## TnT               0.000      0.000  56.254  -1.223    0.226
## log.MPODNA:day    0.234      0.473 117.891   0.495    0.622
```

2.3.3 Summarise all final models (no confounders)

log.dsDNA:

```
## Linear mixed model fit by REML. t-tests use Satterthwaite's method [
## lmerModLmerTest]
## Formula: as.formula(fma)
##    Data: dat
##
## REML criterion at convergence: 1153.8
##
## Scaled residuals:
##     Min      1Q  Median      3Q     Max
## -2.3637 -0.5533 -0.0314  0.5724  3.7483
##
## Random effects:
##  Groups   Name        Variance Std.Dev.
##  ID       (Intercept) 71.38    8.449
##  Residual             18.51    4.303
## Number of obs: 176, groups:  ID, 60
##
## Fixed effects:
##                Estimate Std. Error       df t value Pr(>|t|)
## (Intercept)     66.7472    20.1064 136.4426   3.320  0.00116 **
## log.dsDNA       -4.0577     3.3786 135.7707  -1.201  0.23185
## day              4.3888     5.4901 123.1275   0.799  0.42559
## log.dsDNA:day   -0.5635     0.9180 123.2171  -0.614  0.54044
## ---
## Signif. codes:  0 '***' 0.001 '**' 0.01 '*' 0.05 '.' 0.1 ' ' 1
##
## Correlation of Fixed Effects:
##             (Intr) lg.DNA day
## log.dsDNA   -0.998
## day         -0.652  0.652
## lg.dsDNA:dy  0.659 -0.659 -1.000
```

log.CitH3:

```
## Linear mixed model fit by REML. t-tests use Satterthwaite's method [
## lmerModLmerTest]
## Formula: as.formula(fma)
##    Data: dat
##
## REML criterion at convergence: 1159.7
##
## Scaled residuals:
##     Min      1Q  Median      3Q     Max
## -2.2368 -0.5082 -0.0983  0.5560  3.6214
##
## Random effects:
##  Groups   Name        Variance Std.Dev.
##  ID       (Intercept) 71.99    8.485
##  Residual             18.33    4.282
## Number of obs: 176, groups:  ID, 60
##
## Fixed effects:
##                Estimate Std. Error        df t value Pr(>|t|)
## (Intercept)    42.71058    1.34621 100.61564  31.726   <2e-16 ***
## log.CitH3       0.01753    0.60508 131.34337   0.029   0.9769
## day             0.47309    0.26112 116.76459   1.812   0.0726 .
## log.CitH3:day   0.32679    0.17303 118.72834   1.889   0.0614 .
## ---
## Signif. codes:  0 '***' 0.001 '**' 0.01 '*' 0.05 '.' 0.1 ' ' 1
##
## Correlation of Fixed Effects:
##             (Intr) lg.CH3 day
## log.CitH3   -0.481
## day         -0.332  0.408
## log.CtH3:dy  0.299 -0.597 -0.812
```

log.MPODNA:

```
## Linear mixed model fit by REML. t-tests use Satterthwaite's method [
## lmerModLmerTest]
## Formula: as.formula(fma)
##    Data: dat
##
## REML criterion at convergence: 1161
##
## Scaled residuals:
##     Min      1Q  Median      3Q     Max
## -2.3746 -0.5628 -0.0564  0.5784  3.8636
##
## Random effects:
##  Groups   Name        Variance Std.Dev.
##  ID       (Intercept) 73.49    8.573
##  Residual             18.99    4.358
## Number of obs: 176, groups:  ID, 60
##
## Fixed effects:
##                Estimate Std. Error       df t value Pr(>|t|)
## (Intercept)     42.4667     2.2766 171.9614  18.653   <2e-16 ***
## log.MPODNA      -0.1589     1.4903 137.8116  -0.107   0.9152
## day              1.2610     0.7084 118.4098   1.780   0.0776 .
## log.MPODNA:day   0.2372     0.4734 117.6008   0.501   0.6173
## ---
## Signif. codes:  0 '***' 0.001 '**' 0.01 '*' 0.05 '.' 0.1 ' ' 1
##
## Correlation of Fixed Effects:
##             (Intr) lg.MPODNA day
## log.MPODNA   0.851
## day         -0.293 -0.299
## lg.MPODNA:d -0.339 -0.387     0.973
```

3.1 WMSI

3.1.1 Fit models with IL-8 and with and without NETs variables

dsDNA:

```
iy <- 1

#
# Model with IL-8 only:
fma0 <- paste(Y[iy], "~ IL8 * day + (1 | ID)")
fit0 <- lmer(as.formula(fma0), data=dat, REML = TRUE)
summary(fit0)
```

```
## Linear mixed model fit by REML. t-tests use Satterthwaite's method [
## lmerModLmerTest]
## Formula: as.formula(fma0)
##    Data: dat
##
## REML criterion at convergence: 19.2
##
## Scaled residuals:
##      Min       1Q   Median       3Q      Max
## -2.27563 -0.50157 -0.02009  0.61078  2.41195
##
## Random effects:
##  Groups   Name        Variance Std.Dev.
##  ID       (Intercept) 0.04763  0.2182
##  Residual             0.02958  0.1720
## Number of obs: 177, groups:  ID, 61
##
## Fixed effects:
##               Estimate Std. Error         df t value Pr(>|t|)
## (Intercept)  1.944e+00  4.266e-02  1.333e+02  45.553  < 2e-16 ***
## IL8         -3.732e-04  8.748e-04  1.575e+02  -0.427  0.67019
## day         -3.871e-02  1.279e-02  1.319e+02  -3.026  0.00298 **
## IL8:day      2.028e-05  5.857e-04  1.450e+02   0.035  0.97243
## ---
## Signif. codes:  0 '***' 0.001 '**' 0.01 '*' 0.05 '.' 0.1 ' ' 1
##
## Correlation of Fixed Effects:
##         (Intr) IL8    day
## IL8     -0.629
## day     -0.232  0.179
## IL8:day -0.008 -0.014 -0.866
```

```
#
# Model with IL-8 and NETs variable:
fma1 <- paste(Y[iy], "~ IL8 * day + ", X[3],"* day + (1 | ID)")
fit1 <- lmer(as.formula(fma1), data=dat, REML = TRUE)
summary(fit1)
```

```
## Linear mixed model fit by REML. t-tests use Satterthwaite's method [
## lmerModLmerTest]
## Formula: as.formula(fma1)
```

```
##    Data: dat
##
## REML criterion at convergence: 29.3
##
## Scaled residuals:
##      Min       1Q   Median       3Q      Max
## -2.26152 -0.48801 -0.00323  0.61658  2.39908
##
## Random effects:
##  Groups   Name        Variance Std.Dev.
##  ID       (Intercept) 0.04760  0.2182
##  Residual             0.03003  0.1733
## Number of obs: 177, groups:  ID, 61
##
## Fixed effects:
##                   Estimate Std. Error         df t value Pr(>|t|)
## (Intercept)      1.951e+00  8.573e-02  1.675e+02  22.756   <2e-16 ***
## IL8             -3.331e-04  8.884e-04  1.573e+02  -0.375    0.708
## day             -2.928e-02  3.273e-02  1.251e+02  -0.895    0.373
## log.MPODNA       6.740e-03  5.624e-02  1.540e+02   0.120    0.905
## IL8:day         -1.994e-05  6.000e-04  1.410e+02  -0.033    0.974
## day:log.MPODNA   5.493e-03  1.908e-02  1.243e+02   0.288    0.774
## ---
## Signif. codes:  0 '***' 0.001 '**' 0.01 '*' 0.05 '.' 0.1 ' ' 1
##
## Correlation of Fixed Effects:
##             (Intr) IL8    day    l.MPOD IL8:dy
## IL8         -0.358
## day         -0.360  0.194
## log.MPODNA   0.865 -0.046 -0.316
## IL8:day      0.015 -0.039 -0.498  0.012
## dy:l.MPODNA -0.421  0.134  0.914 -0.438 -0.171
```

```r
#
# Compare both models (does including NETs improve model fit?):
anova(fit0, fit1)
```

```
## Data: dat
## Models:
## fit0: as.formula(fma0)
## fit1: as.formula(fma1)
##      npar     AIC    BIC logLik deviance  Chisq Df Pr(>Chisq)
## fit0    6 -7.6116 11.445 9.8058  -19.612
## fit1    8 -3.7731 21.636 9.8866  -19.773 0.1615  2     0.9224
```

CitH3:

```
#
# Model with IL-8 only:
fma0 <- paste(Y[iy], "~ IL8 * day + (1 | ID)")
fit0 <- lmer(as.formula(fma0), data=dat, REML = TRUE)
summary(fit0)
```

```
## Linear mixed model fit by REML. t-tests use Satterthwaite's method [
## lmerModLmerTest]
## Formula: as.formula(fma0)
##    Data: dat
##
## REML criterion at convergence: 19.2
##
## Scaled residuals:
##      Min       1Q   Median       3Q      Max
## -2.27563 -0.50157 -0.02009  0.61078  2.41195
##
## Random effects:
##  Groups   Name        Variance Std.Dev.
##  ID       (Intercept) 0.04763  0.2182
##  Residual             0.02958  0.1720
## Number of obs: 177, groups:  ID, 61
##
## Fixed effects:
##               Estimate Std. Error         df t value Pr(>|t|)
## (Intercept)  1.944e+00  4.266e-02  1.333e+02  45.553  < 2e-16 ***
## IL8         -3.732e-04  8.748e-04  1.575e+02  -0.427  0.67019
## day         -3.871e-02  1.279e-02  1.319e+02  -3.026  0.00298 **
## IL8:day      2.028e-05  5.857e-04  1.450e+02   0.035  0.97243
## ---
## Signif. codes:  0 '***' 0.001 '**' 0.01 '*' 0.05 '.' 0.1 ' ' 1
##
## Correlation of Fixed Effects:
##         (Intr) IL8    day
## IL8     -0.629
## day     -0.232  0.179
## IL8:day -0.008 -0.014 -0.866
```

```
#
# Model with IL-8 and NETs variable:
fma1 <- paste(Y[iy], "~ IL8 * day + ", X[2],"* day + (1 | ID)")
fit1 <- lmer(as.formula(fma1), data=dat, REML = TRUE)
summary(fit1)
```

```
## Linear mixed model fit by REML. t-tests use Satterthwaite's method [
## lmerModLmerTest]
## Formula: as.formula(fma1)
##    Data: dat
##
## REML criterion at convergence: 32.9
##
## Scaled residuals:
##      Min       1Q   Median       3Q      Max
## -2.24833 -0.50432 -0.00947  0.57981  2.43362
```

```
##
## Random effects:
##  Groups   Name        Variance Std.Dev.
##  ID       (Intercept) 0.04691  0.2166
##  Residual             0.03011  0.1735
## Number of obs: 177, groups:  ID, 61
##
## Fixed effects:
##                  Estimate Std. Error         df t value Pr(>|t|)
## (Intercept)     1.944e+00  4.952e-02  1.544e+02  39.261   <2e-16 ***
## IL8            -3.457e-04  8.810e-04  1.554e+02  -0.392   0.6953
## day            -3.557e-02  1.423e-02  1.253e+02  -2.500   0.0137 *
## log.CitH3      -2.222e-03  2.364e-02  1.476e+02  -0.094   0.9252
## IL8:day         1.229e-04  6.104e-04  1.439e+02   0.201   0.8407
## day:log.CitH3 -3.685e-03  7.160e-03  1.265e+02  -0.515   0.6077
## ---
## Signif. codes:  0 '***' 0.001 '**' 0.01 '*' 0.05 '.' 0.1 ' ' 1
##
## Correlation of Fixed Effects:
##             (Intr) IL8    day    lg.CH3 IL8:dy
## IL8         -0.533
## day         -0.325  0.168
## log.CitH3   -0.505 -0.027  0.253
## IL8:day     -0.062 -0.012 -0.653  0.094
## day:lg.CtH3  0.344 -0.008 -0.423 -0.618 -0.246
```

```r
#
# Compare both models (does including NETs improve model fit?):
anova(fit0, fit1)
```

```
## Data: dat
## Models:
## fit0: as.formula(fma0)
## fit1: as.formula(fma1)
##      npar     AIC    BIC  logLik deviance  Chisq Df Pr(>Chisq)
## fit0    6 -7.6116 11.445  9.8058  -19.612
## fit1    8 -4.1563 21.253 10.0782  -20.156 0.5447  2     0.7616
```

MPODNA:

```r
#
# Model with IL-8 only:
fma0 <- paste(Y[iy], "~ IL8 * day + (1 | ID)")
fit0 <- lmer(as.formula(fma0), data=dat, REML = TRUE)
summary(fit0)
```

```
## Linear mixed model fit by REML. t-tests use Satterthwaite's method [
## lmerModLmerTest]
## Formula: as.formula(fma0)
##    Data: dat
##
## REML criterion at convergence: 19.2
##
## Scaled residuals:
##      Min       1Q   Median       3Q      Max
## -2.27563 -0.50157 -0.02009  0.61078  2.41195
##
## Random effects:
##  Groups   Name        Variance Std.Dev.
##  ID       (Intercept) 0.04763  0.2182
##  Residual             0.02958  0.1720
## Number of obs: 177, groups:  ID, 61
##
## Fixed effects:
##               Estimate Std. Error         df t value Pr(>|t|)
## (Intercept)  1.944e+00  4.266e-02  1.333e+02  45.553  < 2e-16 ***
## IL8         -3.732e-04  8.748e-04  1.575e+02  -0.427  0.67019
## day         -3.871e-02  1.279e-02  1.319e+02  -3.026  0.00298 **
## IL8:day      2.028e-05  5.857e-04  1.450e+02   0.035  0.97243
## ---
## Signif. codes:  0 '***' 0.001 '**' 0.01 '*' 0.05 '.' 0.1 ' ' 1
##
## Correlation of Fixed Effects:
##         (Intr) IL8    day
## IL8     -0.629
## day     -0.232  0.179
## IL8:day -0.008 -0.014 -0.866
```

```r
#
# Model with IL-8 and NETs variable:
fma1 <- paste(Y[iy], "~ IL8 * day + ", X[3],"* day + (1 | ID)")
fit1 <- lmer(as.formula(fma1), data=dat, REML = TRUE)
summary(fit1)
```

```
## Linear mixed model fit by REML. t-tests use Satterthwaite's method [
## lmerModLmerTest]
## Formula: as.formula(fma1)
##    Data: dat
##
## REML criterion at convergence: 29.3
##
## Scaled residuals:
##      Min       1Q   Median       3Q      Max
## -2.26152 -0.48801 -0.00323  0.61658  2.39908
```

```
##
## Random effects:
##  Groups   Name        Variance Std.Dev.
##  ID       (Intercept) 0.04760  0.2182
##  Residual             0.03003  0.1733
## Number of obs: 177, groups:  ID, 61
##
## Fixed effects:
##                  Estimate Std. Error         df t value Pr(>|t|)
## (Intercept)     1.951e+00  8.573e-02  1.675e+02  22.756   <2e-16 ***
## IL8            -3.331e-04  8.884e-04  1.573e+02  -0.375    0.708
## day            -2.928e-02  3.273e-02  1.251e+02  -0.895    0.373
## log.MPODNA      6.740e-03  5.624e-02  1.540e+02   0.120    0.905
## IL8:day        -1.994e-05  6.000e-04  1.410e+02  -0.033    0.974
## day:log.MPODNA  5.493e-03  1.908e-02  1.243e+02   0.288    0.774
## ---
## Signif. codes:  0 '***' 0.001 '**' 0.01 '*' 0.05 '.' 0.1 ' ' 1
##
## Correlation of Fixed Effects:
##             (Intr) IL8    day    l.MPOD IL8:dy
## IL8         -0.358
## day         -0.360  0.194
## log.MPODNA   0.865 -0.046 -0.316
## IL8:day      0.015 -0.039 -0.498  0.012
## dy:l.MPODNA -0.421  0.134  0.914 -0.438 -0.171
```

```
#
# Compare both models (does including NETs improve model fit?):
anova(fit0, fit1)
```

```
## Data: dat
## Models:
## fit0: as.formula(fma0)
## fit1: as.formula(fma1)
##      npar     AIC    BIC logLik deviance  Chisq Df Pr(>Chisq)
## fit0    6 -7.6116 11.445 9.8058  -19.612
## fit1    8 -3.7731 21.636 9.8866  -19.773 0.1615  2     0.9224
```

3.2 GLS

3.2.1 Fit models with IL-8 and with and without NETs variables

dsDNA:

```
iy <- 2
#
# Model with IL-8 only:
fma0 <- paste(Y[iy], "~ IL8 * day + (1 | ID)")
fit0 <- lmer(as.formula(fma0), data=dat, REML = TRUE)
summary(fit0)
```

```
## Linear mixed model fit by REML. t-tests use Satterthwaite's method [
## lmerModLmerTest]
## Formula: as.formula(fma0)
##    Data: dat
##
## REML criterion at convergence: 573.2
##
## Scaled residuals:
##      Min       1Q   Median       3Q      Max
## -2.42878 -0.57319  0.07332  0.47303  2.45248
##
## Random effects:
##  Groups   Name        Variance Std.Dev.
##  ID       (Intercept) 3.498    1.870
##  Residual             1.400    1.183
## Number of obs: 142, groups:  ID, 51
##
## Fixed effects:
##               Estimate Std. Error         df t value Pr(>|t|)
## (Intercept)  -9.353837   0.369160  99.958929 -25.338   <2e-16 ***
## IL8           0.008612   0.007291 114.985589   1.181    0.240
## day          -0.165389   0.107521 101.080157  -1.538    0.127
## IL8:day      -0.006230   0.005173 109.771837  -1.204    0.231
## ---
## Signif. codes:  0 '***' 0.001 '**' 0.01 '*' 0.05 '.' 0.1 ' ' 1
##
## Correlation of Fixed Effects:
##         (Intr) IL8    day
## IL8     -0.598
## day     -0.166  0.127
## IL8:day -0.037  0.032 -0.890
```

```
#
# Model with IL-8 and NETs variable:
fma1 <- paste(Y[iy], "~ IL8 * day + ", X[1],"* day + (1 | ID)")
fit1 <- lmer(as.formula(fma1), data=dat, REML = TRUE)
summary(fit1)
```

```
## Linear mixed model fit by REML. t-tests use Satterthwaite's method [
## lmerModLmerTest]
## Formula: as.formula(fma1)
##    Data: dat
```

```
##
## REML criterion at convergence: 569.9
##
## Scaled residuals:
##     Min      1Q  Median      3Q     Max
## -2.4019 -0.5430  0.0166  0.5060  2.4358
##
## Random effects:
##  Groups   Name        Variance Std.Dev.
##  ID       (Intercept) 3.472    1.863
##  Residual             1.400    1.183
## Number of obs: 142, groups:  ID, 51
##
## Fixed effects:
##                  Estimate Std. Error         df t value Pr(>|t|)
## (Intercept)    -18.197162   6.565691 115.664118  -2.772   0.0065 **
## IL8              0.011826   0.007630 118.986698   1.550   0.1238
## day              0.530841   1.817657  99.928838   0.292   0.7709
## log.dsDNA        1.473858   1.092774 114.639118   1.349   0.1801
## IL8:day         -0.008564   0.005695 107.737168  -1.504   0.1356
## day:log.dsDNA  -0.111323   0.308062 100.319714  -0.361   0.7186
## ---
## Signif. codes:  0 '***' 0.001 '**' 0.01 '*' 0.05 '.' 0.1 ' ' 1
##
## Correlation of Fixed Effects:
##              (Intr) IL8    day    lg.DNA IL8:dy
## IL8          -0.326
## day          -0.656  0.178
## log.dsDNA    -0.998  0.294  0.656
## IL8:day       0.103 -0.016  0.188 -0.105
## dy:lg.dsDNA   0.645 -0.167 -0.998 -0.646 -0.242
```

```r
#
# Compare both models (does including NETs improve model fit?):
anova(fit0, fit1)
```

```
## Data: dat
## Models:
## fit0: as.formula(fma0)
## fit1: as.formula(fma1)
##      npar    AIC    BIC  logLik deviance  Chisq Df Pr(>Chisq)
## fit0    6 563.39 581.13 -275.70   551.39
## fit1    8 565.04 588.69 -274.52   549.04 2.3491  2      0.309
```

CitH3:

```
#
# Model with IL-8 only:
fma0 <- paste(Y[iy], "~ IL8 * day + (1 | ID)")
fit0 <- lmer(as.formula(fma0), data=dat, REML = TRUE)
summary(fit0)
```

```
## Linear mixed model fit by REML. t-tests use Satterthwaite's method [
## lmerModLmerTest]
## Formula: as.formula(fma0)
##    Data: dat
##
## REML criterion at convergence: 573.2
##
## Scaled residuals:
##      Min       1Q   Median       3Q      Max
## -2.42878 -0.57319  0.07332  0.47303  2.45248
##
## Random effects:
##  Groups   Name        Variance Std.Dev.
##  ID       (Intercept) 3.498    1.870
##  Residual             1.400    1.183
## Number of obs: 142, groups:  ID, 51
##
## Fixed effects:
##              Estimate Std. Error         df t value Pr(>|t|)
## (Intercept)  -9.353837   0.369160  99.958929 -25.338   <2e-16 ***
## IL8           0.008612   0.007291 114.985589   1.181    0.240
## day          -0.165389   0.107521 101.080157  -1.538    0.127
## IL8:day      -0.006230   0.005173 109.771837  -1.204    0.231
## ---
## Signif. codes:  0 '***' 0.001 '**' 0.01 '*' 0.05 '.' 0.1 ' ' 1
##
## Correlation of Fixed Effects:
##         (Intr) IL8    day
## IL8     -0.598
## day     -0.166  0.127
## IL8:day -0.037  0.032 -0.890
```

```
#
# Model with IL-8 and NETs variable:
fma1 <- paste(Y[iy], "~ IL8 * day + ", X[2],"* day + (1 | ID)")
fit1 <- lmer(as.formula(fma1), data=dat, REML = TRUE)
summary(fit1)
```

```
## Linear mixed model fit by REML. t-tests use Satterthwaite's method [
## lmerModLmerTest]
## Formula: as.formula(fma1)
##    Data: dat
##
## REML criterion at convergence: 576
##
## Scaled residuals:
##      Min       1Q   Median       3Q      Max
## -2.42421 -0.58664  0.09788  0.51099  2.37228
```

```
##
## Random effects:
##  Groups   Name        Variance Std.Dev.
##  ID       (Intercept) 3.335    1.826
##  Residual             1.414    1.189
## Number of obs: 142, groups:  ID, 51
##
## Fixed effects:
##                  Estimate Std. Error         df t value Pr(>|t|)
## (Intercept)     -9.364441   0.442323 122.205513 -21.171   <2e-16 ***
## IL8              0.009089   0.007341 115.765246   1.238    0.218
## day             -0.114289   0.115008  93.910696  -0.994    0.323
## log.CitH3       -0.020984   0.195700 111.515220  -0.107    0.915
## IL8:day         -0.003587   0.005473 111.677900  -0.655    0.514
## day:log.CitH3   -0.071351   0.056043  96.890058  -1.273    0.206
## ---
## Signif. codes:  0 '***' 0.001 '**' 0.01 '*' 0.05 '.' 0.1 ' ' 1
##
## Correlation of Fixed Effects:
##              (Intr) IL8    day    lg.CH3 IL8:dy
## IL8          -0.557
## day          -0.265  0.151
## log.CitH3    -0.563  0.106  0.218
## IL8:day      -0.132  0.049 -0.678  0.169
## day:lg.CtH3   0.394 -0.085 -0.348 -0.651 -0.318
```

```r
#
# Compare both models (does including NETs improve model fit?):
anova(fit0, fit1)
```

```
## Data: dat
## Models:
## fit0: as.formula(fma0)
## fit1: as.formula(fma1)
##      npar    AIC    BIC  logLik deviance  Chisq Df Pr(>Chisq)
## fit0    6 563.39 581.13 -275.70   551.39
## fit1    8 564.18 587.83 -274.09   548.18 3.2079  2     0.2011
```

MPODNA:

```
#
# Model with IL-8 only:
fma0 <- paste(Y[iy], "~ IL8 * day + (1 | ID)")
fit0 <- lmer(as.formula(fma0), data=dat, REML = TRUE)
summary(fit0)
```

```
## Linear mixed model fit by REML. t-tests use Satterthwaite's method [
## lmerModLmerTest]
## Formula: as.formula(fma0)
##    Data: dat
##
## REML criterion at convergence: 573.2
##
## Scaled residuals:
##      Min       1Q   Median       3Q      Max
## -2.42878 -0.57319  0.07332  0.47303  2.45248
##
## Random effects:
##  Groups   Name        Variance Std.Dev.
##  ID       (Intercept) 3.498    1.870
##  Residual             1.400    1.183
## Number of obs: 142, groups:  ID, 51
##
## Fixed effects:
##               Estimate Std. Error         df t value Pr(>|t|)
## (Intercept)  -9.353837   0.369160  99.958929 -25.338   <2e-16 ***
## IL8           0.008612   0.007291 114.985589   1.181    0.240
## day          -0.165389   0.107521 101.080157  -1.538    0.127
## IL8:day      -0.006230   0.005173 109.771837  -1.204    0.231
## ---
## Signif. codes:  0 '***' 0.001 '**' 0.01 '*' 0.05 '.' 0.1 ' ' 1
##
## Correlation of Fixed Effects:
##         (Intr) IL8    day
## IL8     -0.598
## day     -0.166  0.127
## IL8:day -0.037  0.032 -0.890
```

```
#
# Model with IL-8 and NETs variable:
fma1 <- paste(Y[iy], "~ IL8 * day + ", X[3],"* day + (1 | ID)")
fit1 <- lmer(as.formula(fma1), data=dat, REML = TRUE)
summary(fit1)
```

```
## Linear mixed model fit by REML. t-tests use Satterthwaite's method [
## lmerModLmerTest]
## Formula: as.formula(fma1)
##    Data: dat
##
## REML criterion at convergence: 575
##
## Scaled residuals:
##      Min       1Q   Median       3Q      Max
## -2.40737 -0.57721  0.02842  0.50315  2.42584
```

```
##
## Random effects:
##  Groups   Name        Variance Std.Dev.
##  ID       (Intercept) 3.461    1.860
##  Residual             1.432    1.197
## Number of obs: 142, groups:  ID, 51
##
## Fixed effects:
##                  Estimate Std. Error         df t value Pr(>|t|)
## (Intercept)     -9.409813   0.645296 133.200007 -14.582   <2e-16 ***
## IL8              0.009463   0.007474 116.442080   1.266    0.208
## day             -0.045881   0.245665  92.114696  -0.187    0.852
## log.MPODNA      -0.022992   0.426381 113.588958  -0.054    0.957
## IL8:day         -0.006753   0.005328 105.418516  -1.268    0.208
## day:log.MPODNA   0.074168   0.140455  91.172674   0.528    0.599
## ---
## Signif. codes:  0 '***' 0.001 '**' 0.01 '*' 0.05 '.' 0.1 ' ' 1
##
## Correlation of Fixed Effects:
##              (Intr) IL8    day    l.MPOD IL8:dy
## IL8          -0.313
## day          -0.306  0.191
## log.MPODNA    0.817  0.045 -0.256
## IL8:day      -0.025 -0.007 -0.549 -0.021
## dy:l.MPODNA -0.389  0.137  0.890 -0.406 -0.171
```

```r
#
# Compare both models (does including NETs improve model fit?):
anova(fit0, fit1)
```

```
## Data: dat
## Models:
## fit0: as.formula(fma0)
## fit1: as.formula(fma1)
##      npar    AIC    BIC  logLik deviance  Chisq Df Pr(>Chisq)
## fit0    6 563.39 581.13 -275.70   551.39
## fit1    8 567.08 590.73 -275.54   551.08 0.3128  2     0.8552
```

3.3 EF

3.3.1 Fit models with IL-8 and with and without NETs variables

dsDNA:

```
iy <- 3
#
# Model with IL-8 only:
fma0 <- paste(Y[iy], "~ IL8 * day + (1 | ID)")
fit0 <- lmer(as.formula(fma0), data=dat, REML = TRUE)
summary(fit0)
```

```
## Linear mixed model fit by REML. t-tests use Satterthwaite's method [
## lmerModLmerTest]
## Formula: as.formula(fma0)
##    Data: dat
##
## REML criterion at convergence: 1164.1
##
## Scaled residuals:
##     Min      1Q  Median      3Q     Max
## -2.3679 -0.5495 -0.0311  0.5294  3.8537
##
## Random effects:
##  Groups   Name        Variance Std.Dev.
##  ID       (Intercept) 73.26    8.559
##  Residual             19.13    4.374
## Number of obs: 174, groups:  ID, 60
##
## Fixed effects:
##              Estimate Std. Error        df t value Pr(>|t|)
## (Intercept)  42.27168    1.40252 106.80399  30.140  < 2e-16 ***
## IL8           0.01346    0.02362 136.77577   0.570 0.569654
## day           1.26096    0.33909 122.11709   3.719 0.000304 ***
## IL8:day      -0.01652    0.01570 129.71692  -1.052 0.294720
## ---
## Signif. codes:  0 '***' 0.001 '**' 0.01 '*' 0.05 '.' 0.1 ' ' 1
##
## Correlation of Fixed Effects:
##         (Intr) IL8    day
## IL8     -0.522
## day     -0.151  0.117
## IL8:day -0.044  0.060 -0.874
```

```
#
# Model with IL-8 and NETs variable:
fma1 <- paste(Y[iy], "~ IL8 * day + ", X[1],"* day + (1 | ID)")
fit1 <- lmer(as.formula(fma1), data=dat, REML = TRUE)
summary(fit1)
```

```
## Linear mixed model fit by REML. t-tests use Satterthwaite's method [
## lmerModLmerTest]
## Formula: as.formula(fma1)
##    Data: dat
```

```
##
## REML criterion at convergence: 1154.9
##
## Scaled residuals:
##     Min      1Q  Median      3Q     Max
## -2.3247 -0.5486 -0.0249  0.5762  3.6977
##
## Random effects:
##  Groups   Name        Variance Std.Dev.
##  ID       (Intercept) 71.59    8.461
##  Residual             19.11    4.372
## Number of obs: 174, groups:  ID, 60
##
## Fixed effects:
##                  Estimate Std. Error         df t value Pr(>|t|)
## (Intercept)    66.052564  21.682104 140.554995   3.046  0.00277 **
## IL8             0.004001   0.024758 141.926948   0.162  0.87186
## day             4.069929   6.035342 122.579214   0.674  0.50136
## log.dsDNA      -3.963336   3.603892 139.246779  -1.100  0.27334
## IL8:day        -0.003230   0.018089 128.169961  -0.179  0.85857
## day:log.dsDNA  -0.498548   1.028117 123.106259  -0.485  0.62860
## ---
## Signif. codes:  0 '***' 0.001 '**' 0.01 '*' 0.05 '.' 0.1 ' ' 1
##
## Correlation of Fixed Effects:
##             (Intr) IL8    day    lg.DNA IL8:dy
## IL8         -0.335
## day         -0.623  0.173
## log.dsDNA   -0.998  0.304  0.623
## IL8:day      0.000  0.033  0.344 -0.003
## dy:lg.dsDNA  0.615 -0.165 -0.998 -0.616 -0.391
```

```r
#
# Compare both models (does including NETs improve model fit?):
anova(fit0, fit1)
```

```
## Data: dat
## Models:
## fit0: as.formula(fma0)
## fit1: as.formula(fma1)
##      npar    AIC    BIC  logLik deviance  Chisq Df Pr(>Chisq)
## fit0    6 1164.1 1183.1 -576.07   1152.1
## fit1    8 1164.7 1190.0 -574.34   1148.7 3.4656  2     0.1768
```

CitH3:

```
#
# Model with IL-8 only:
fma0 <- paste(Y[iy], "~ IL8 * day + (1 | ID)")
fit0 <- lmer(as.formula(fma0), data=dat, REML = TRUE)
summary(fit0)
```

```
## Linear mixed model fit by REML. t-tests use Satterthwaite's method [
## lmerModLmerTest]
## Formula: as.formula(fma0)
##    Data: dat
##
## REML criterion at convergence: 1164.1
##
## Scaled residuals:
##     Min      1Q  Median      3Q     Max
## -2.3679 -0.5495 -0.0311  0.5294  3.8537
##
## Random effects:
##  Groups   Name        Variance Std.Dev.
##  ID       (Intercept) 73.26    8.559
##  Residual             19.13    4.374
## Number of obs: 174, groups:  ID, 60
##
## Fixed effects:
##              Estimate Std. Error        df t value Pr(>|t|)
## (Intercept)  42.27168    1.40252 106.80399  30.140  < 2e-16 ***
## IL8           0.01346    0.02362 136.77577   0.570 0.569654
## day           1.26096    0.33909 122.11709   3.719 0.000304 ***
## IL8:day      -0.01652    0.01570 129.71692  -1.052 0.294720
## ---
## Signif. codes:  0 '***' 0.001 '**' 0.01 '*' 0.05 '.' 0.1 ' ' 1
##
## Correlation of Fixed Effects:
##         (Intr) IL8    day
## IL8     -0.522
## day     -0.151  0.117
## IL8:day -0.044  0.060 -0.874
```

```
#
# Model with IL-8 and NETs variable:
fma1 <- paste(Y[iy], "~ IL8 * day + ", X[2],"* day + (1 | ID)")
fit1 <- lmer(as.formula(fma1), data=dat, REML = TRUE)
summary(fit1)
```

```
## Linear mixed model fit by REML. t-tests use Satterthwaite's method [
## lmerModLmerTest]
## Formula: as.formula(fma1)
##    Data: dat
##
## REML criterion at convergence: 1158.2
##
## Scaled residuals:
##     Min      1Q  Median      3Q     Max
## -2.1791 -0.5082 -0.1038  0.5547  3.4840
```

```
##
## Random effects:
##  Groups   Name        Variance Std.Dev.
##  ID       (Intercept) 71.44    8.452
##  Residual             18.53    4.304
## Number of obs: 174, groups:  ID, 60
##
## Fixed effects:
##                 Estimate Std. Error        df t value Pr(>|t|)
## (Intercept)     42.55399    1.53584 131.19796  27.707   <2e-16 ***
## IL8              0.01088    0.02327 134.36207   0.468   0.6408
## day              0.91025    0.36613 117.50170   2.486   0.0143 *
## log.CitH3       -0.10710    0.61499 127.76683  -0.174   0.8620
## IL8:day         -0.02670    0.01599 128.06594  -1.670   0.0974 .
## day:log.CitH3    0.40410    0.18134 116.56776   2.228   0.0278 *
## ---
## Signif. codes:  0 '***' 0.001 '**' 0.01 '*' 0.05 '.' 0.1 ' ' 1
##
## Correlation of Fixed Effects:
##             (Intr) IL8    day    lg.CH3 IL8:dy
## IL8         -0.467
## day         -0.235  0.119
## log.CitH3   -0.433 -0.008  0.226
## IL8:day     -0.095  0.068 -0.665  0.110
## day:lg.CtH3  0.295 -0.029 -0.410 -0.607 -0.251
```

```
#
# Compare both models (does including NETs improve model fit?):
anova(fit0, fit1)
```

```
## Data: dat
## Models:
## fit0: as.formula(fma0)
## fit1: as.formula(fma1)
##      npar    AIC    BIC  logLik deviance  Chisq Df Pr(>Chisq)
## fit0    6 1164.1 1183.1 -576.07   1152.1
## fit1    8 1160.9 1186.1 -572.43   1144.9 7.2797  2    0.02626 *
## ---
## Signif. codes:  0 '***' 0.001 '**' 0.01 '*' 0.05 '.' 0.1 ' ' 1
```

MPODNA:

```r
#
# Model with IL-8 only:
fma0 <- paste(Y[iy], "~ IL8 * day + (1 | ID)")
fit0 <- lmer(as.formula(fma0), data=dat, REML = TRUE)
summary(fit0)
```

```
## Linear mixed model fit by REML. t-tests use Satterthwaite's method [
## lmerModLmerTest]
## Formula: as.formula(fma0)
##    Data: dat
##
## REML criterion at convergence: 1164.1
##
## Scaled residuals:
##     Min      1Q  Median      3Q     Max
## -2.3679 -0.5495 -0.0311  0.5294  3.8537
##
## Random effects:
##  Groups   Name        Variance Std.Dev.
##  ID       (Intercept) 73.26    8.559
##  Residual             19.13    4.374
## Number of obs: 174, groups:  ID, 60
##
## Fixed effects:
##              Estimate Std. Error        df t value Pr(>|t|)
## (Intercept)  42.27168    1.40252 106.80399  30.140  < 2e-16 ***
## IL8           0.01346    0.02362 136.77577   0.570 0.569654
## day           1.26096    0.33909 122.11709   3.719 0.000304 ***
## IL8:day      -0.01652    0.01570 129.71692  -1.052 0.294720
## ---
## Signif. codes:  0 '***' 0.001 '**' 0.01 '*' 0.05 '.' 0.1 ' ' 1
##
## Correlation of Fixed Effects:
##         (Intr) IL8    day
## IL8     -0.522
## day     -0.151  0.117
## IL8:day -0.044  0.060 -0.874
```

```r
#
# Model with IL-8 and NETs variable:
fma1 <- paste(Y[iy], "~ IL8 * day + ", X[3],"* day + (1 | ID)")
fit1 <- lmer(as.formula(fma1), data=dat, REML = TRUE)
summary(fit1)
```

```
## Linear mixed model fit by REML. t-tests use Satterthwaite's method [
## lmerModLmerTest]
## Formula: as.formula(fma1)
##    Data: dat
##
## REML criterion at convergence: 1160.6
##
## Scaled residuals:
##     Min      1Q  Median      3Q     Max
## -2.3257 -0.5293 -0.0370  0.5308  3.7631
```

```
##
## Random effects:
##  Groups   Name        Variance Std.Dev.
##  ID       (Intercept) 73.62    8.580
##  Residual             19.33    4.396
## Number of obs: 174, groups:  ID, 60
##
## Fixed effects:
##                  Estimate Std. Error        df t value Pr(>|t|)
## (Intercept)      41.91346    2.42972 166.16265  17.250   <2e-16 ***
## IL8               0.01623    0.02401 135.99073   0.676   0.5001
## day               1.84933    0.84503 115.51875   2.188   0.0306 *
## log.MPODNA       -0.20446    1.51027 132.69138  -0.135   0.8925
## IL8:day          -0.01841    0.01597 125.83384  -1.153   0.2512
## day:log.MPODNA    0.36886    0.49387 115.64223   0.747   0.4566
## ---
## Signif. codes:  0 '***' 0.001 '**' 0.01 '*' 0.05 '.' 0.1 ' ' 1
##
## Correlation of Fixed Effects:
##              (Intr) IL8    day    l.MPOD IL8:dy
## IL8          -0.330
## day          -0.300  0.185
## log.MPODNA    0.814 -0.029 -0.272
## IL8:day      -0.010  0.035 -0.485  0.008
## dy:l.MPODNA -0.365  0.149  0.910 -0.393 -0.145
```

```r
#
# Compare both models (does including NETs improve model fit?):
anova(fit0, fit1)
```

```
## Data: dat
## Models:
## fit0: as.formula(fma0)
## fit1: as.formula(fma1)
##      npar    AIC    BIC  logLik deviance  Chisq Df Pr(>Chisq)
## fit0    6 1164.1 1183.1 -576.07   1152.1
## fit1    8 1167.5 1192.8 -575.76   1151.5 0.6123  2     0.7363
```

Software

```
## R version 4.0.2 (2020-06-22)
## Platform: x86_64-apple-darwin17.0 (64-bit)
## Running under: macOS Catalina 10.15.6
##
## Matrix products: default
## BLAS:   /Library/Frameworks/R.framework/Versions/4.0/Resources/lib/libRblas.dylib
## LAPACK: /Library/Frameworks/R.framework/Versions/4.0/Resources/lib/libRlapack.dylib
##
## locale:
## [1] en_US.UTF-8/en_US.UTF-8/en_US.UTF-8/C/en_US.UTF-8/en_US.UTF-8
##
## attached base packages:
## [1] stats     graphics  grDevices utils     datasets  methods   base
##
## other attached packages:
## [1] lmerTest_3.1-2  lme4_1.1-23     Matrix_1.2-18   Hmisc_4.4-0
## [5] ggplot2_3.3.2   Formula_1.2-3   survival_3.1-12 lattice_0.20-41
## [9] haven_2.3.1
##
## loaded via a namespace (and not attached):
##  [1] statmod_1.4.34      tidyselect_1.1.0    xfun_0.16
##  [4] purrr_0.3.4         splines_4.0.2       colorspace_1.4-1
##  [7] vctrs_0.3.3         generics_0.0.2      htmltools_0.5.0
## [10] yaml_2.2.1          base64enc_0.1-3     rlang_0.4.7
## [13] nloptr_1.2.2.1      pillar_1.4.6        foreign_0.8-80
## [16] glue_1.4.2          withr_2.2.0         RColorBrewer_1.1-2
## [19] jpeg_0.1-8.1        lifecycle_0.2.0     stringr_1.4.0
## [22] munsell_0.5.0       gtable_0.3.0        htmlwidgets_1.5.1
## [25] evaluate_0.14       latticeExtra_0.6-29 knitr_1.29
## [28] forcats_0.5.0       htmlTable_1.13.3    Rcpp_1.0.5
## [31] acepack_1.4.1       readr_1.3.1         scales_1.1.1
## [34] backports_1.1.9     checkmate_2.0.0     gridExtra_2.3
## [37] hms_0.5.3           png_0.1-7           digest_0.6.25
## [40] stringi_1.4.6       dplyr_1.0.2         numDeriv_2016.8-1.1
## [43] grid_4.0.2          tools_4.0.2         magrittr_1.5
## [46] tibble_3.0.3        cluster_2.1.0       crayon_1.3.4
## [49] pkgconfig_2.0.3     MASS_7.3-51.6       ellipsis_0.3.1
## [52] data.table_1.13.0   minqa_1.2.4         rmarkdown_2.2
## [55] rstudioapi_0.11     boot_1.3-25         R6_2.4.1
## [58] rpart_4.1-15        nlme_3.1-148        nnet_7.3-14
## [61] compiler_4.0.2
```
